# An update on *Aenocyon dirus* in the interior of North America: new records, radiocarbon dates, ZooMS spectra, and isotopic data for an iconic late Pleistocene carnivore

**DOI:** 10.7717/peerj.19219

**Published:** 2025-04-11

**Authors:** Matthew G. Hill, Christopher C. Widga, Todd A. Surovell, Kurt M. Wilson, Sarah A. Allaun, McKenna L. Litynski, Jason Titcomb

**Affiliations:** 1 World Languages and Cultures, Iowa State University, Ames, Iowa, United States; 2 Department of Geosciences, Pennsylvania State University, University Park, Pennsylvania, United States; 3 Department of Anthropology, University of Wyoming, Laramie, Wyoming, United States; 4 Department of Anthropology, Lawrence University, Appleton, Wisconsin, United States; 5 Office of the State Archaeologist of Colorado, Denver, Colorado, United States; 6 St. Augustine Lighthouse & Maritime Museum, St. Augustine, Florida, United States

**Keywords:** Quaternary, Pleistocene, Canidae, Dire wolf, Vertebrate taphonomy, Paleoecology, Extinction

## Abstract

*Aenocyon dirus* played a crucial role as a predator in late Quaternary megafaunal communities throughout southern North America. This article presents two new occurrences of the species from southwest Iowa on the eastern Great Plains, updates the Peccary Cave record in the southern Ozark Highlands, and amends the fossil record of the species. In southern North America, there are 166 occurrences of *A. dirus*, spanning Marine Isotope Stage (MIS) 2-19, with at least two-thirds (*n* = 112) of the occurrences dating to MIS 2-3 (11,600–57,000 cal B.P.). *A. dirus* fossils are found across this region, with notable concentrations in California, Florida, the Ozark Highlands, and broadly across the southern Great Plains. Consideration of *Canis* specimens from the lead region (covering contiguous parts of Illinois, Wisconsin, and Iowa) previously identified as *Canis mississippiensis* (and sometimes synonymized with *A. dirus* or *C. lupus*) reveals they are actually *C. lupus*. The terminal extinction of *A. dirus* occurred sometime after 12,800 cal B.P.

The Iowa finds, consisting of a radius and a partial cranium, are the first records for the state. Zooarchaeology by mass spectrometry confirms these records, as well as the Peccary Cave record, are *A. dirus*, as opposed to *C. lupus*. The Iowa specimens are directly dated to 29,040–28,410 cal B.P. and 14,325–14,075 cal B.P., while Peccary Cave is dated to 25,350–21,405 cal B.P. These results place *A. dirus* in the interior of southern North America before, during, and after the Last Glacial Maximum (26,500–19,000 cal B.P.). Stable nitrogen isotope (δ^15^N) values of bone collagen from the younger of the two Iowa records suggest this individual did not regularly compete for prey with *Smilodon fatalis* during the Bølling-Allerød Chronozone (14,640–12,850 cal B.P.). To the south, at Peccary Cave, considerations of prey size, prey abundance, and isotopic data strongly suggest *Platygonus compressus* was the focal prey species.

## Introduction

Radiocarbon ages, stable isotopes, ancient DNA, taphonomic findings, and occurrences of taxa are necessary for documenting the timing and taxonomic ordering of late Quaternary megafaunal die-offs, changes in species’ interactions and life histories, shifts in diet and trophic connections, and biogeographic adjustments. However, owing to research priorities within disciplines, researcher preferences, taphonomic controls on fossil storage, and trophic controls on fossil abundance, the supply of empirical information is geographically and taxonomically patchy. As Stuart put it, “The need is for much more high quality data, not more debate based on imperfect evidence” (Stuart, 2015, 338), a sentiment echoed more recently by Meltzer’s remark that “Linking change to cause will require additional data” (Meltzer, 2020, 28559). For example, extinction of *Mammuthus* and *Mammut* is reasonably grounded in the Great Lakes region and in Beringia (Enk et al., 2016; Guthrie, 2004; Guthrie, 2006; Mann et al., 2013; Widga et al., 2021; Widga et al., 2017; Yansa & Adams, 2012; Zazula et al., 2014); *Oreamnos harringtoni* and *Nothrotheriops* in the Southwest (Mead et al., 1986; Steadman et al., 2005; Thompson et al., 1980); *Equus* in the Great Basin (Jenkins et al., 2012); and *Equus*, *Bison*, and *Ovibos* in Beringia (Campos et al., 2010; Guthrie, 2004; Guthrie, 2006; Heintzman et al., 2017; Mann et al., 2013; Shapiro et al., 2004). The same cannot be said for many other herbivores, from medium-sized taxa such as *Castoroides* and *Mylohyus fossilis* to megafauna such as *Camelops*, *Bootherium*, and *Cervalces*.

The situation with large carnivores is more acute, and three iconic taxa—*Smilodon fatalis*, *Aenocyon dirus*, and *Arctodus simus—*illustrate this point. Direct radiometric ages for *S. fatalis* and *A. dirus* in southern North America total around 100 and 80 dates, respectively. Except for one date for *S. fatalis* from Iowa (Hill & Easterla, 2023, Table 1) and three dates from two localities for *A. dirus* from Wyoming and Tennessee (Perri et al., 2021, S1), all dates are from Rancho La Brea (RLB), California (Fuller et al., 2014; Fuller et al., 2015, Table 2; Fuller et al., 2020; O’Keefe et al., 2023, Table 1; O’Keefe, Fet & Harris, 2009). Twenty-six direct ages for *A. simus* are dispersed between Beringia, the Great Basin, the central Great Plains, and the eastern United States, including five (on the same individual) from Sheriden Cave, Ohio (Dansie & Jerrems, 2005, Table 1; Schubert, 2010, Tables 2 and 3). Three results are from RLB (Fuller et al., 2015, Table 2) and one is from McKittrick, California (Fox-Dobbs et al., 2014, Table 1). Fresh data on large carnivore ecology and biogeography are plainly needed for many studies concerning late Quaternary megafaunal extinctions.

On the eastern Great Plains and Ozark Highlands, *S. fatalis*, *A. dirus*, and *A. simus* occur at 35 localities, primarily in and around the Ozarks. This includes 16 *A. simus* (Richards, Churcher & Turnbull, 1996, Figure 1; Schubert, 2010), 14 *A. dirus* (Czaplewski, Rogers & Russell, 2018, 100; Dundas, 1999, Figure 1), and five *S. fatalis* (Hill & Easterla, 2023, Figure 1). Direct radiocarbon dates are available for three *A. simus* records and one *S. fatalis* record.

**Figure 1:**
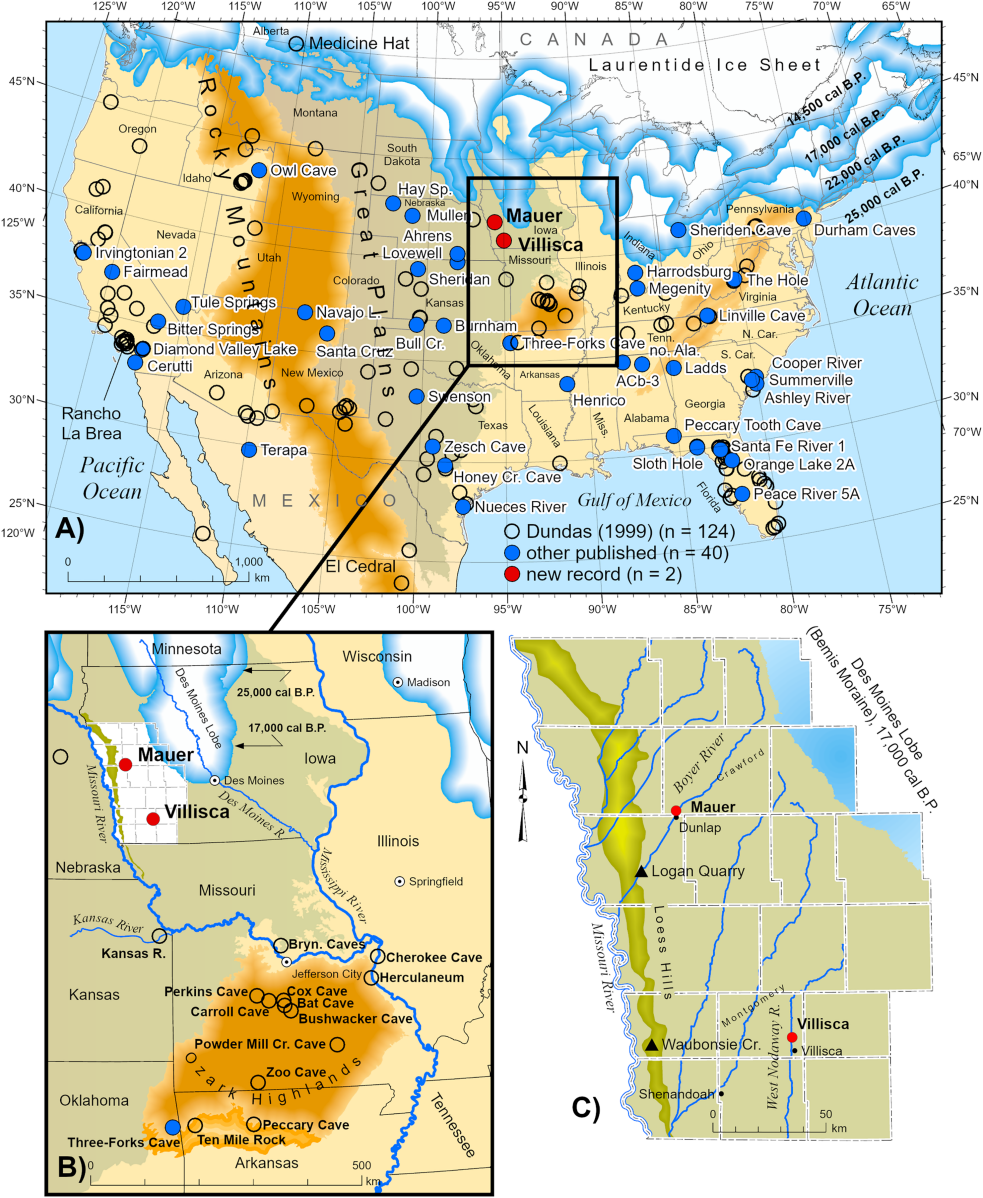
Distribution of *Aenocyon dirus* records in southern North America. “*Aenocyon dirus*” includes *A. dirus*, cf. *A. dirus*, *A*. cf. *dirus*, and aff. *A. dirus*. Laurentide Ice Sheet deglaciation isochrones are shown at 22,000 cal B.P. (Last Glacial Maximum), 17,000, and 14,500 cal B.P. (~Bølling-Allerød) (Dalton et al., 2023, SOM). Some state and locality names are abbreviated on the map to reduce clutter. Data S7. New records (*n* = 42). ACb-3, Alabama (Ebersole & Ebersole, 2011; Jacquemin et al., 2016, 11). Northern Alabama (Ebersole & Ebersole, 2011, 11). Albert Ahrens, Hay Springs, and Mullen, Nebraska (Tedford, Wang & Taylor, 2009). Ashley River, South Carolina (Albright et al., 2019). Bitter Springs playa, California (Reynolds & Reynolds, 1994). Bull Creek, Oklahoma (Carlson & Bement, 2017). Burnham, Oklahoma (Czaplewski, 2003). Cerutti, California (Holen et al., 2017). Cooper River, South Carolina (Sanders, 2002). Henrico dikes, Arkansas (Baghai-Riding et al., 2017). Diamond Valley Lake, California (Springer et al., 2009). Durham Caves, Pennsylvania (Tucci, 1986; Tucci, 1987). Fairmead and Irvingtonian 2, California (Tedford, Wang & Taylor, 2009, 132; Trayler et al., 2015). Harrodsburg Crevice, Indiana (Munson, Parmalee & Guilday, 1980; Parmalee, Munson & Guilday, 1978; Smith & Polly, 2013). Honey Creek Cave, Texas (Toomey, 1994). Ladds, Georgia (Nowak, 2002, 107, 128). Linville Cave, Tennessee (Franklin & Dean, 2006). Lovewell Reservoir, Kansas (Holen, 2006; Holen, Corner & Mandel, 1995). Mauer, Iowa (this study). Megenity Peccary Cave, Indiana (Nowak, 2002, 127; Richards, 1995, 90; Richards & Whitaker, 1997, 149). Navajo Lake, New Mexico (Morgan & Lucas, 2005). Nueces River, Texas (Sagebiel, 2022). Orange Lake 2A, Florida (Morgan & Seymour, 1997). Owl Cave, Idaho (Miller & Dort, 1978). Peace River 5A, Florida (Hulbert, Morgan & Kerner, 2009). Peccary Tooth Cave, Florida (Gillette, 1979). Santa Cruz, New Mexico (Morgan & Lucas, 2005). Santa Fe River 1, Florida (Morgan & Seymour, 1997). Swenson, Texas (Lundelius, 2022). Sheridan (ghost town), Kansas (this study). Sheriden Cave, Ohio (Perri et al., 2021, S1). Sloth Hole, Florida (Hemmings, 2005). Summerville, South Carolina (Albright et al., 2019). Térapa, Mexico (Short et al., 2021). The Hole, West Virginia (Garton & Grady, 2018). Three-Forks Cave, Oklahoma (Czaplewski, Rogers & Russell, 2018). Tule Springs, Nevada (Scott & Springer, 2016). Villisca, Iowa (this study). Zesch Cave, Texas (Sagebiel, 2010). Illustration credit: Matthew G. Hill.

In this article, we report two new *A. dirus* records from southwestern Iowa on the east-central Great Plains. One is a partial cranium from the Mauer sand-and-gravel pit on the Boyer River near Dunlap, while the second is a radius from the West Nodaway River near Villisca (Fig. 1). The taxon’s presence in Iowa is not unexpected; however, these specimens represent the first formal reports of *A. dirus* for the state. We also report on the *A. dirus* collection from Peccary Cave, Arkansas, in the southern Ozarks (Fig. 1). Several direct dates constrain the age of this material. Although the presence of *A. dirus* at Peccary Cave was noted decades ago (Quinn, 1972), the content and character of the sample were unknown. Zooarchaeology by mass spectrometry (ZooMS) confirms the Iowa specimens and the Peccary Cave material are *A. dirus*. Additionally, in updating the spatiotemporal distribution of *A. dirus* records, we resolve the longstanding uncertainty surrounding the find-spot and taxonomic relationships of the enigmatic large canid, *Canis mississippiensis* (Allen, 1876). Finally, we develop paleozoologic and radiometric evidence that offers new insights into the body size, timing of extinction, biogeography, and diet of *A. dirus* in southern North America.

## Localities

### Mauer, Iowa

Joe and Buertess Beals collected this partial *A. dirus* cranium in August 1959 from the now-flooded Mauer sand-and-gravel pit on the Boyer River in Crawford County, Iowa (Figs. 1B, 1C). The find-spot is 0.3 km north of the small town of Dunlap (WGS84 41.86N, 95.60W). The specimen predates the Last Glacial Maximum (LGM) (26,500–19,000 cal B.P.) (Clark et al., 2009) by about 2,500 years, and is currently the oldest directly dated *A. dirus* east of the Rocky Mountains. It is curated at the Sanford Museum and Planetarium (SMP), Cherokee, Iowa (catalog number 149-59-Z). The SMP’s paleontological locality number is 13CF4.

### Villisca, Iowa

The *A. dirus* radius was collected by Jim McClarnon in May 2019 in shallow water resting on the bed of the West Nodaway River, 6 km north of the small town of Villisca, Montgomery County, Iowa (WGS84 40.97N, 94.98W) (Figs. 1B, 1C). Direct dating suggests it likely eroded from an upstream exposure of Noah Creek Formation, an extensive, thick unit of late Pleistocene sands-and-gravels (~17,000–13,000 cal B.P.) (Bettis & Kemmis, 1996, 23–24; Quade, 2006; Tassier-Surine et al., 2012). The specimen is curated in the Paleontological Repository, Department of Earth and Environmental Sciences, University of Iowa (SUI), Iowa City, Iowa. The catalog number is SUI-149737.

### Peccary Cave, Arkansas

**Figure 2:**
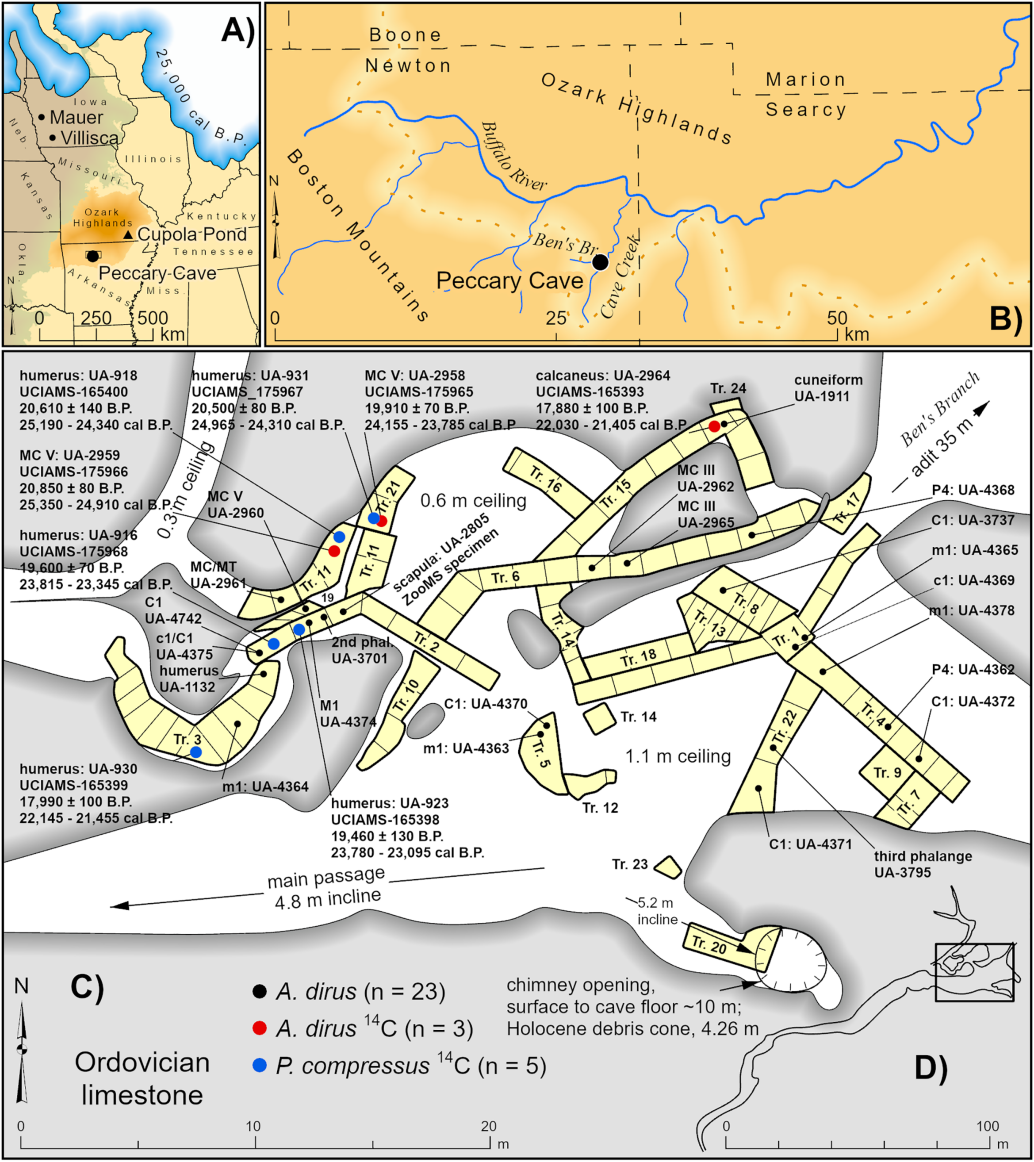
Location and plan view maps of Peccary Cave. (A) Regional location. (B) Local location. (C) Plan view of main chamber, excavation trench nos. 1–24, distribution of *A. dirus* remains, and directly dated *A. dirus* and *P. compressus* specimens. (D) Plan view of cave. Trench no. 9 is located between Trench nos. 2 and 11, and Trench nos. 11 and 14 are not contiguous. Specimens missing excavation square information (*n* = 5) are mapped near the middle of the trench. MC, Metacarpal. MT, Metatarsal. *A. dirus* dates (Table 6). *P. compressus* dates (Wilson & Hill, 2020, Table 1). Illustration credit: Matthew G. Hill.

Peccary Cave is situated in Ordovician limestone in the southern Ozarks, near the confluence of Ben’s Branch and Cave Creek, a tributary to the Buffalo River, in Newton County, northwestern Arkansas (WGS84 35.92N, 92.97W) (Figs. 2A, 2B). Excavations in 1967–1969 (University of Arkansas, UA) and 1971–1972 (University of Iowa) yielded a large, taxonomically diverse collection of extinct and extant vertebrate remains. The identified small mammals are curated at SUI, while UA retains the balance of the collection. Only the last four digits of UA accession numbers are used in the text and illustrations because the full numbers are cumbersome (*e.g*., UA-67-300-075-4370 = UA-4370).

The original cave entrance, through which an adit (horizontal tunnel) was dug to facilitate paleontological investigations, was located 35 m northeast of the main chamber, flush with the valley-floor of Ben’s Branch, and measured 9 m wide and 2 m high (Quinn, 1972, 91) (Fig. 2C). Today this entrance is 8 m above the valley-floor. The main chamber measures 25 m east/west and 10 m north/south. It extends in a southwesterly direction into a relatively long, narrow tunnel (Fig. 2D), and in a northwesterly direction into a smaller room measuring 10 m east/west and 5 m north/south. Several low passages branch from the smaller room and encircle limestone columns. Ceiling height ranges from about a meter to places where the floor and ceiling nearly converge. The bone-bearing stratum extends to a depth of 50–60 cm and consists of a highly churned mixture of bones and teeth, *Platygonus compressus* droppings, *Mesodon* shells, fine-grained alluvium (clay, silt, and sand), and angular rock (≤50 mm in diameter) (Davis, 1967, 1969; Quinn, 1972; Semken, 1984, 407). The churning is likely the result of a slow rate of sedimentation and bioturbation by *P. compressus* and *Erethizon dorsatum*. Given that *Mesodon* does not inhabit caves (Hubricht, 1985, 42–45), it undoubtedly was transported as flotsam in floodwaters that breached the cave entrance and flowed into the main chamber.

Previously, Davis’ (1973) work on the reptiles and amphibians, along with the studies by Semken and colleagues on the small mammals (Graham & Semken, 1976; Hallberg, Semken & Davis, 1974; Megivern, 1982; Semken, 1984; Semken, Graham & Stafford, 2010; Stafford & Semken, 1990; Stafford et al., 1999), accounted for the substantive research on the collection. More recently, Wilson and Hill (Wilson, 2017; Wilson & Hill, 2020) analyzed the *P. compressus* assemblage. It includes 100 adult and subadult animals, as well as 14 fetuses in various stages of development. Of the other extinct medium- and large-sized taxa, the *A. dirus* sample is the largest, totaling 26 identified specimens representing at least 3 individuals. This sample is described and discussed here for the first time. Three dates on *A. dirus* and five dates on *P. compressus* suggest most, if not all, remains of these taxa accumulated during the LGM.

## Methods

For the time periods and locations under consideration here, three large canid species are relevant to identification: *A. dirus*, *C. lupus*, and *C. rufus* (Agenbroad & Mead, 1986; Czaplewski & Mead, 1990; Meachen, Brannick & Fry, 2016; Nowak, 1979; Nowak, 2002; Nowak, 2003, Fig. 9.7; Perri et al., 2021; Tedford, Wang & Taylor, 2009; Tomiya & Meachen, 2018; Wilson & Rutledge, 2021). However, distinguishing bones and teeth of these taxa is challenging, particularly when dealing with isolated and fragmentary specimens. The use of modern reference skeletons, including comparative metric data, is therefore mandatory as a diagnostic aid. Thus, Hill and Widga directly compared each fossil specimen to analogous bones and teeth in two wild Alaskan *C. lupus* skeletons: an adult male and a young adult female. Metric comparisons were also made with 44 *C. lupus* skeletons from the Great Lakes region (Wisconsin, Minnesota, and the Upper Peninsula of Michigan) and 299 *C. lupus* and 100 *C. rufus* skulls from across North America (Goldman, 1964). These are referred to as the ‘Great Lakes’ and ‘North America’ samples, respectively (Data S1, S2).

*A. dirus* from RLB serves as the analytical baseline for researchers working on this taxon, and measurements on this material were integrated into analyses when possible, along with data from other locations. In order to reduce inter-analyst variation (Breslawski & Byers, 2015; Lyman & Van Pool, 2009), only original primary data from RLB collected by several researchers was used (*i.e*., Koper, 2013; Nigra, 1946; Nigra & Lance, 1946; Nowak, 1979; Stock & Lance, 1948) (Data S3, S4). *A. dirus* calcanea and radii in the Field Museum of Natural History collections, Chicago, were also measured. Comparative *A. dirus* data exclusive of RLB are provided in Data S5.

Univariate and bivariate plots were used to display general morphological differences in *C. lupus*, *C. rufus*, and *A. dirus* bones and teeth. Most plots were produced using summary statistics provided in Table S1. These results were calculated using PAST (ver. 4.13) (Hammer, Harper & Ryan, 2001).

Zooarchaeology by mass spectrometry (Buckley et al., 2009; Richter et al., 2022) was also performed on several samples to augment the results of morphological diagnoses. The procedure leverages known locations of peptide markers to determine or verify the taxonomic identity of bone. To this end, partial MALDI-TOF MS spectra of the tryptic digests of collagen were generated for the fossil samples for comparison with *C. lupus* and *C. latrans* spectra. Analytical methods are described in Article S2.

AMS radiocarbon ages (and accompanying stable isotope results) were obtained to determine the geological age of specimens. Measurement of gelatinized collagen extracted from a sample of the Mauer cranium using an acid-base-acid (ABA) sequence (Talamo et al., 2021, 63) was conducted at Aeon Laboratories, Tucson, Arizona. Aeon sample preparation and pretreatment protocols for ABA collagen extraction are available in Article S3. For Villisca and three Peccary Cave specimens, measurement of gelatinized collagen purified by ultrafiltration was performed at the Keck Carbon Cycle AMS Facility, University of California, Irvine (UCIAMS), employing the facility’s standard procedure (Fuller et al., 2014, 86). OxCal 4.4 (Ramsey, 1995; Ramsey, 2001) and IntCal20 (Reimer et al., 2020) were used to calibrate the measured radiocarbon ages, including previously published ones we cite.

Samples of four right *P. compressus* humeri from Peccary Cave were processed in the Stable Isotope Laboratory, Department of the Earth, Atmosphere, and Climate, Iowa State University, to complement previously published isotopic data on five directly dated samples (Wilson & Hill, 2020, Table 1). Collagen extraction followed the “whole bone” procedure (Sealy et al., 2014). Approximately 1 gram of whole cortical bone was removed from each specimen. Sample surfaces were cleaned by placing them in a sonic bath for two 20-min intervals, with ultrapure water rinses between each interval. Demineralization was initiated by submerging the samples in 0.25 M hydrochloric acid, which was replaced every two days until a translucent supernatant was observed. The samples were then rinsed to neutrality with ultrapure water. To remove humic acids, they were treated with 0.125 M sodium hydroxide for 6 h before being rinsed to neutrality. Next, the samples were solubilized in 4 mL of 0.25 M HCl at 85 °C for 24 h. The solubilized samples were then filtered using 9 mL Ezee filter separators (60–90 μm) and finally lyophilized, yielding freeze-dried collagen.

Samples were measured *via* a Thermo Finnigan Delta Plus XL mass spectrometer in continuous flow mode connected to a Costech Elemental Analyzer. Reference standards (Caffeine (USGS-62), Caffeine (IAEA-600), Cellulose (IAEA-CH-3) and Acetanilide (laboratory standard)) were used for isotopic corrections, and to assign the data to the appropriate isotopic scale. Corrections were done using a regression method and isotope results are reported in parts per thousand (per mil, ‰). Percent concentration (%) was calculated using the peak area of the sample. The combined uncertainty (analytical uncertainty and average correction factor) for δ^13^C is ± 0.16‰ (VPDB) and δ^15^N is ± 0.09‰ (Air), respectively.

The inventory of *A. dirus* in southern North America was updated to situate the Iowa and Peccary Cave fossils in a spatiotemporal context. Southern North America, as defined here, encompasses the conterminous United States and extends from southern Canada to northern Mexico (~25–50°N, ~75–125°W). Each record in this area reported by Dundas (1999) was checked against more recent literature for taxonomic, chronologic, and paleozoologic updates. Using the Neotoma Paleoecology Database (Williams et al., 2018) as a starting point, a literature survey combined with an online search was conducted to locate new records of specimens identified as ‘*A. dirus*, cf. *A. dirus*, *A*. cf. *dirus*, and aff. *A. dirus’*. Records were assigned to Marine Isotope Stage bins (MIS) (Lisiecki & Raymo, 2005) to simplify reporting of inferred or confirmed ages (*e.g*., “Rancholabrean” [=MIS 2-7], “Sangamonian” [=MIS 4-5]). Undoubtedly, we missed some new and updated records.

Several tables include specimens with Central Missouri State University (CMS or CMSU) catalog numbers, as referenced in various publications by Oscar Hawksley. The Hawksley collection is now housed at the Illinois State Museum.

Nomenclature for maxillary and mandibular teeth uses uppercase and lowercase letters, respectively, to distinguish them in the text, tables, and figures.

## Results

### Systematic Paleontology

Class Mammalia (Linnaeus, 1758)

Order Carnivora (Bowdich, 1821)

Family Canidae (Fischer von Waldheim, 1817)

Genus *Aenocyon* (Merriam, 1918)

*Aenocyon dirus* (Leidy, 1858)

dire wolf

### Referred material

*Villisca*: radius, SUI-149737.

*Mauer*: cranium, SMP 140-59-Z.

*Peccary Cave*: humerus, UA 67-300-015-1132; cuneiform, UA 67-300-240-1911; scapula, UA 65-158-036-2805; metacarpal V, UA 67-300-254-2958; metacarpal V, UA 67-300-051-2959; metacarpal V, UA 67-300-175-2960; metacarpal/metatarsal V, 6 UA 7-300-127-2961; metacarpal III, UA 67-300-194-2962; calcaneus, UA 67-300-239-2964; metacarpal III, UA 67-300-198-2965; second phalange, UA 65-158-014-3701; C1, UA 67-300-115-3737; third phalange, UA 67-300-182-3795; P4, UA 67-300-300-4362; m1, UA 67-300-30?-4363; m1, UA 67-300-018-4364; m1, UA 65-158-019-4365; P4, UA 67-300-210-4368; c1, UA 67-300-075-4369; C1, UA 67-300-034-4370; C1, UA 67-300-184-4371; C1, UA 67-300-360-4372; M1, UA 65-158-036-4374; C1/c1, UA 67-300-002-4375; m1, UA 67-300-281-4378; C1, UA 65-158-018-4742.

### Descriptions

**Figure 3:**
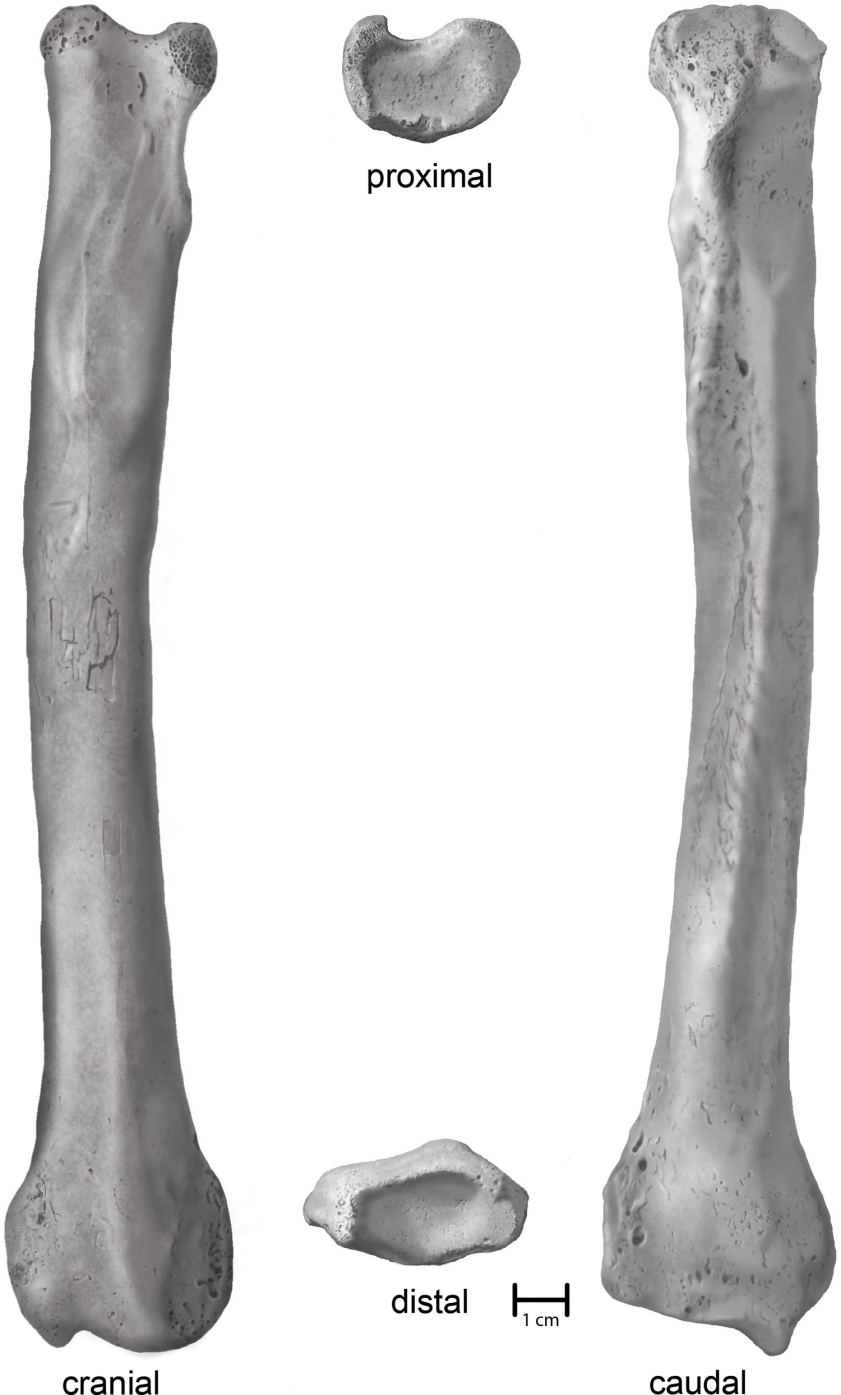
Shaded line illustration of the Villisca *Aenocyon dirus* radius. Illustration credit: Assata Caldwell.

*Villisca*. An anatomically complete left radius (Fig. 3). Union of the proximal and distal articular epiphyses is complete. Physical condition and morphological integrity are good. Fluvial transport *via* traction and saltation has exposed cancellous bone on the margins of the proximal end as well as slightly rounded or polished topographic highs. A small patch (2 cm × 2 cm) of cortical cracking and flaking on the midshaft on the cranial surface suggests a period of subaerial exposure earlier in its taphonomic history.

**Figure 4:**
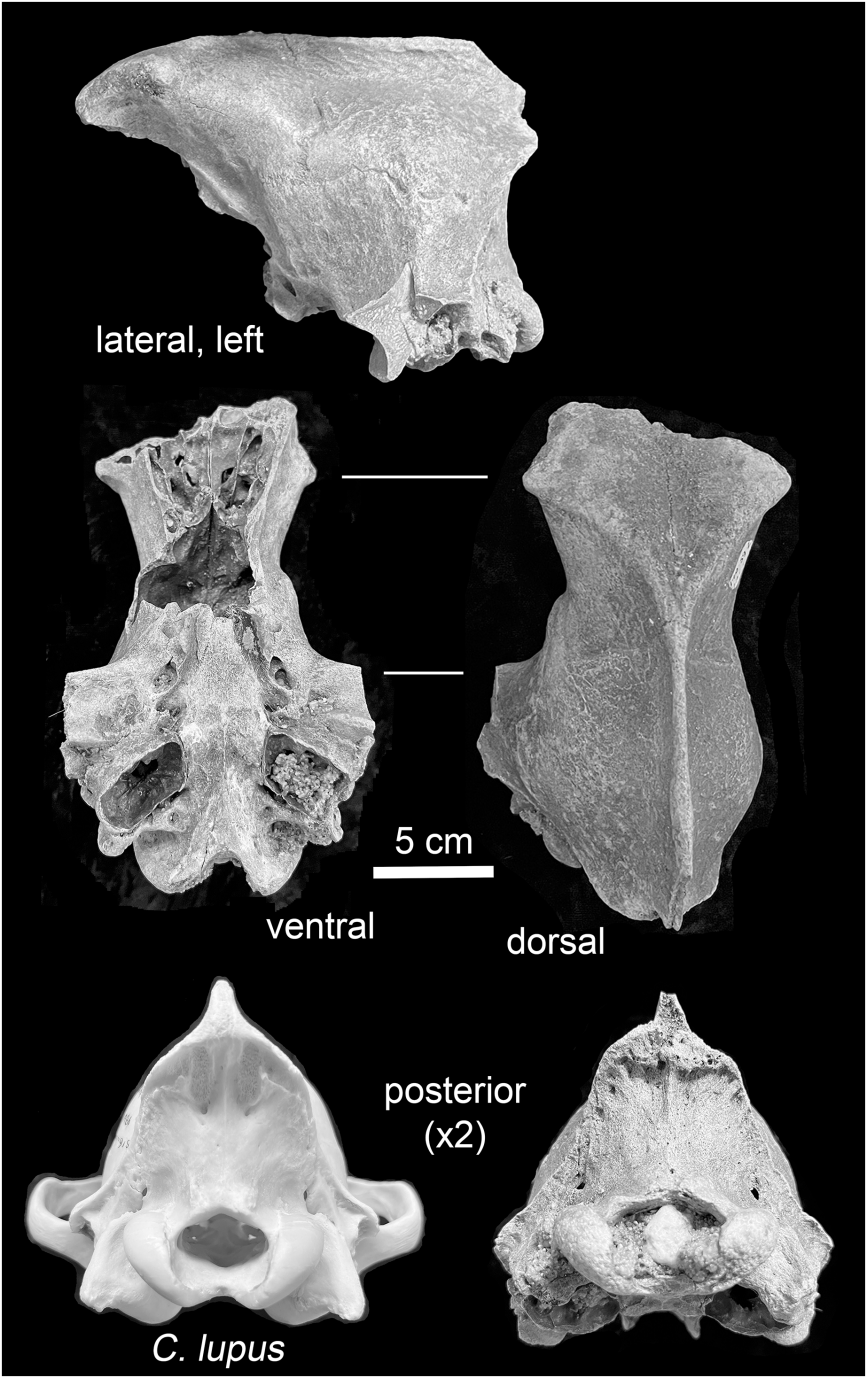
Views of the Mauer *Aenocyon dirus* cranium. Glaciogenic alluvium is visible in the left auditory bulla and foramen magnum. The posterior view of a young adult female Alaskan *C. lupus* cranium is included to highlight the morphological difference in the occipital regions of the taxa. Photograph credit: Matthew G. Hill.

*Mauer*. The preserved portion, a neurocranium, is in fair physical condition (Fig. 4). The foramen magnum and left auditory bulla are partially filled with coarse sand, and the foramen magnum also with a subrounded pebble. These particles are likely traces of the glaciogenic alluvium that stored the specimen in secondary context until discovery. Uniform staining of the fracture edges and intact portions indicate separation of the preserved portion and other cranial parts occurred prior to exposure and recovery. Intact elements include the frontals, parietals, temporals, occipital, basisphenoid, and pterygoids. The external occipital protuberance of the sagittal crest is missing. The zygomatic arches are represented by broken nubs. Both external acoustic meatus are present; the fragile outer (inferior) surfaces of the tympanic bulla are missing. Following a system to describe suture closure in *Homo sapiens* crania (Milner & Boldsen, 2013, 12), the fronto-parietal and parieto-squamosal sutures are partially obliterated, while the parieto-supraoccipital, interfrontal, and basioccipital-basisphenoid sutures are obliterated.

**Figure 5:**
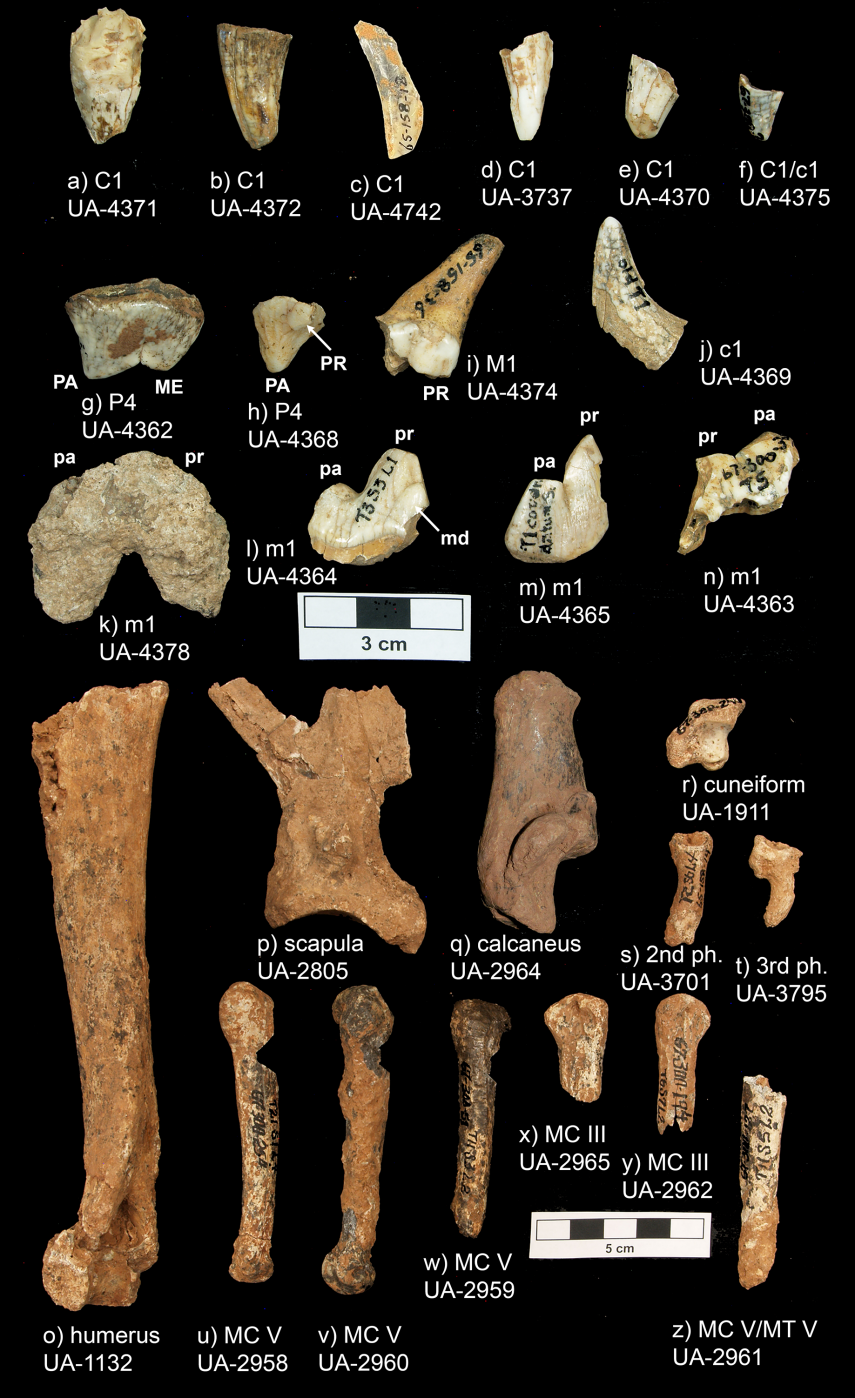
Peccary Cave *Aenocyon dirus* specimens. MC, Metacarpal. MT, Metatarsal. ME, Metacone. PA, Paracone. PR, Protocone (= dueterocone). pa, Paraconid. pr, Protoconid. md, Metaconid. Photograph credit: Matthew G. Hill.

*Peccary Cave*. The assemblage includes 14 complete teeth or tooth fragments and 12 postcranial specimens. These specimens are registered in Table 1 and shown in Fig. 5. Overall, rodent gnawing and calcium carbonate (CaCO3) accretion have significantly impacted the morphological and metric integrity of the specimens, and have also obscured cortical surfaces. This characterization applies to the entire faunal collection. Many long bones and metapodials have been sculpted to cylinders, carpals and tarsals to amorphous nodules, and teeth to crowns (*sensu* Ball & Davis, 1993, 124). *E. dorsatum*, a notorious gnawer, is inferred to be the main culprit (Curtis & Kozicky, 1944, 138; Labanoski, 2017; Pokines et al., 2017, 51; Woods, 1973, 4). Mineral accretion ranges from small, isolated patches and nodules to clumped masses that encase entire specimens.

**Table 1:** Peccary Cave *Aenocyon dirus* inventory.

UA Acc. no.	no. 2	Fig. 5	Trench, Square, Level	Element, Portion, Side	Comments
67-300-184	4371	a	Tr. 22, Sq. 4, Lv. 2	C1, distal	
67-300-360	4372	b	Tr. 4, Sq. 19 thru 21, Lv. 2 ft to 3 ft	C1, distal (buccal “tip”), right	Sliver of buccal crown, blunted from wear
65-158-018	4742	c	Tr. 2, Sq. 7, Lv. 3	C1, distal (buccal “tip”), right	Blunted from wear
67-300-115	3737	d	Tr. 8, Sq. 5, Lv. 3	C1, distal (medial “tip”), left	
67-300-034	4370	e	Tr. 5, Sq. xx, Lv. 1	C1, distal (“tip”)	Blunted from wear
67-300-002	4375	f	Tr. 2, Sq. 7, Lv. 3	C1/cl, distal (“tip”)	
67-300-300	4362	g	Tr. 4, Sq. xx, Lv. 2	P4, complete, left	13.2 mm × 26.0 mm (buccolingual breadth × mesiodistal length)
67-300-210	4368	h	Tr. 6, Sq. 12, Lv. 1	P4, mesiolingual, right	
65-158-036	4374	i	Tr. 2, Sq. 5 thru 7, Lv. 1	M1, distolingual, left	Width of talonid 13.9 mm
67-300-075	4369	j	Tr. 1, Sq. xx, Lv. xx	c1, distal (“tip”), left	
67-300-281	4378	k	Tr. 4, Sq. 2 & 3, Lv. 2 ft to 3 ft	m1, complete, right	~35.7 mm mesiodistal length
67-300-018	4364	l	Tr. 3, Sq. 3, Lv. 1	m1, mesiobuccal, right	Worn main cusps dentine exposed
65-158-019	4365	m	Tr. 1, Sq. offset, Lv. xx	m1, mesiolingual, left	
67-300-30?	4363	n	Tr. 5, Sq. xx, Lv. xx	m1, mesial, right	Distal paraconid/mesial protoconid
67-300-015	1132	o	Tr. 3, Sq. 1, Lv. 2	Humerus, proximal, left	^14^C and ZooMS failed; old fracture
65-158-036	2805	p	Tr. 2, Sq. 5 thru 7, Lv. 1	Scapula, proximal, right	ZooMS sample
67-300-239	2964	q	Tr. 15, Sq. 7, Lv. 1	Calcaneus, complete, left	UCIAMS-165393; ZooMS failed.
67-300-240	1911	r	Tr. 15, Sq. 7, Lv. 2	Cuneiform, complete, right	
65-158-014	3701	s	Tr. 2, Sq. 6, Lv. 4	Second phalange, complete	Greatest length 32.5 mm
67-300-182	3795	t	Tr. 22, Sq. 3, Lv. 2	Third phalange, complete	Greatest length 26.9 mm
67-300-254	2958	u	Tr. 21, Sq. 1, Lv. 4	Metacarpal V, complete, right	UCIAMS-175965; ZooMS failed
67-300-175	2960	v	Tr. 19, Sq. 1, Lv. xx	Metacarpal V, complete, right	
67-300-051	2959	w	Tr. 11, Sq. 3, Lv. 2	Metacarpal V, proximal, right	UCIAMS-175966; ZooMS failed
67-300-198	2965	x	Tr. 6, Sq. 8, Lv. 2	Metacarpal III, proximal, right	Old fracture
67-300-194	2962	y	Tr. 6, Sq. 7, Lv. 2	Metacarpal III, proximal, right	
67-300-127	2961	z	Tr. 11, Sq. 5, Lv. 2	Metacarpal/tarsal V, shaft, left	Recent fracture proximal

### Morphological diagnoses

**Figure 6:**
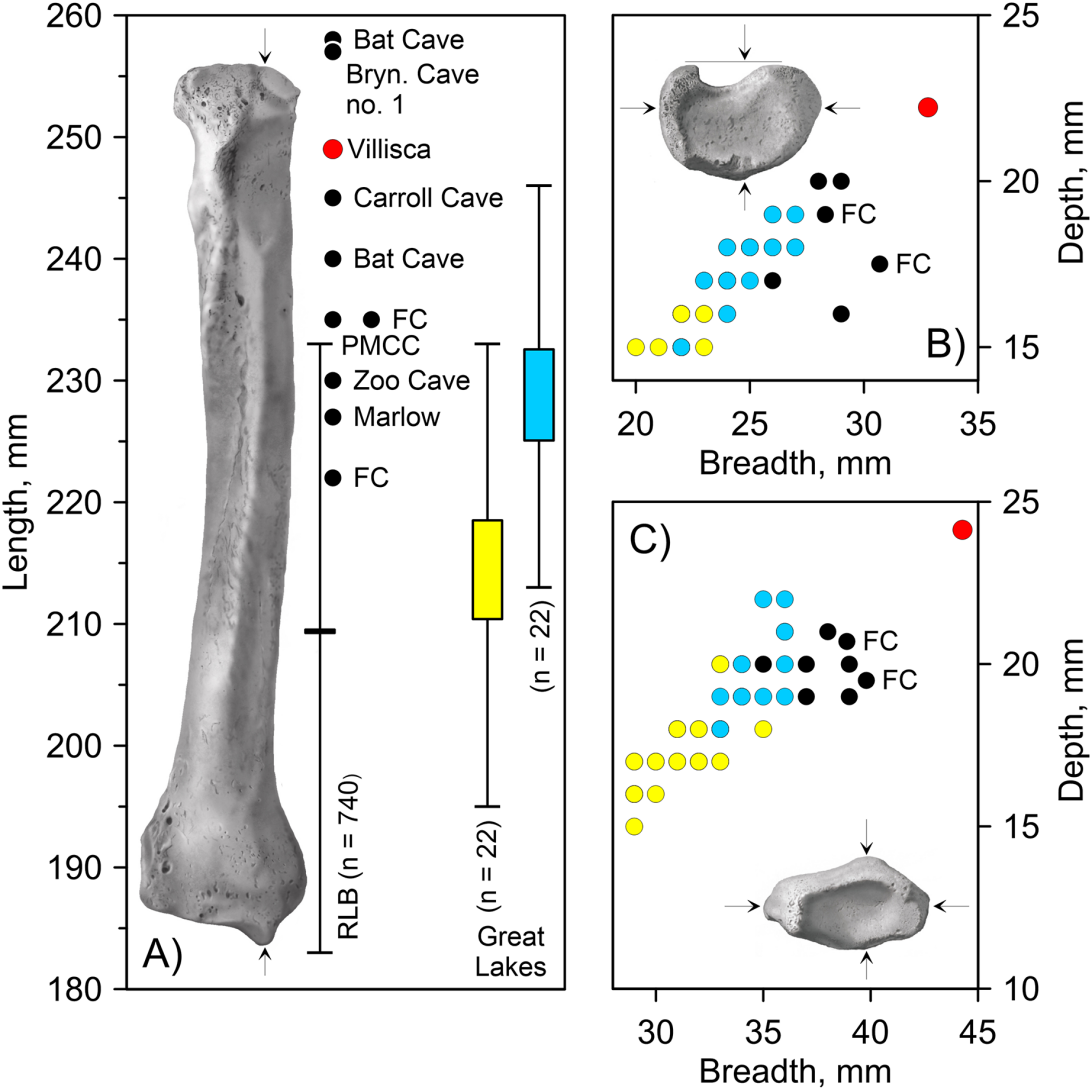
Univariate and bivariate arrays of radius measurements on *Canis lupus* and *Aenocyon dirus*. (A) Greatest length. (B) Proximal end. (C) Distal end. Results for larger samples are displayed as 95% confidence interval box plots with range whiskers. *A. dirus* uses black symbology, except for Villisca (red). Yellow/blue symbology are *C. lupus* females and males, respectively. FC, Friesenhahn Cave. PMCC, Powder Mill Creek Cave. RLB, Rancho La Brea. Unlabeled *A. dirus* data points in the bivariate arrays are Rancho La Brea. Labeling is minimized to reduce clutter, including multiple occurrences of identical *C. lupus* articular end measurements in the bivariate arrays. Table 2. Table S1. Data S1. Illustration credit: Matthew G. Hill.

*Villisca radius*. The length of the radius falls within the range of *A. dirus* from several caves in Missouri (Table 2), all of which surpass the longest example in a sample of 740 specimens from RLB by 1–2 cm (Fig. 6A). In this attribute, it exceeds *C. lupus*. Bivariate arrangements of articular end measurements show similar separation of the taxa (Figs. 6B, 6C). The Missouri specimens lack analogous measurements to be displayed in these plots; however, the available measurements are similar to those in the Villisca specimen (Table 2). Also, the middle groove on the cranial aspect of the distal end, which houses the tendon for the *m. extensor carpi radialis* (Evans, 1993, 191), is shallow and broad, similar to that described in *A. dirus* at RLB and dissimilar to that in *C. lupus* (Merriam, 1912, 237). These comparisons coupled with its overall robustness and relatively faint middle groove are the basis for assigning the Villisca radius to *A. dirus*.

**Table 2:** Measurements on *Aenocyon dirus* radii.

		Attribute, mm						
Record	Cat. no.	GL	PB	PD	DB	DD	MB	MD
Villisca, Iowa	SUI-149737	249.0	32.8	22.2	44.3	24.1	23.4	16.5
Bat Cave, Missouri	–	240.0	–	–	–	–	–	–
Bat Cave, Missouri	–	258.0	–	–	–	–	–	–
Brynjulfson Cave no. 1, Missouri	–	257.0	31.5	–	42.5	–	–	–
Brynjulfson Cave no. 2, Missouri	–	–	–	–	44.5	–	–	–
Carroll Cave, Missouri^1^	CMS-14	245.0	30.7	–	42.6	–	–	–
Friesenhahn Cave, Texas^2^	UNSM-12592	235.0	30.7	17.5	39.8	19.5	20.5	14.2
Friesenhahn Cave, Texas^3^	–	222.0	28.3	19.0	38.9	20.7	20.6	13.2
Marlow, Oklahoma	USNM 10278	227.0	–	–	–	–	–	–
Powder Mill Creek Cave, Missouri	P-249	236.0	31.6	–	40.9	–	–	–
Zoo Cave, Missouri	CMSU-573.2	230.0	–	–	–	–	–	–
Rancho La Brea, California	PM-12490	210.0	29.0	20.0	37.0	19.0	–	–
	PM-12490	–	–	–	37.0	20.0	–	–
	PM-12490	–	–	–	38.0	21.0	–	–
	PM-12490	–	–	–	39.0	20.0	–	–
	PM-12490	–	–	–	34.0	20.0	–	–
	PM-12490	–	–	–	37.0	20.0	–	–
	PM-12490	–	–	–	36.0	20.0	–	–
	PM-12490	–	–	–	35.0	20.0	–	–
	PM-12490	–	–	–	39.0	19.0	–	–
	PM-12490	–	–	–	36.0	20.0	–	–
	PM-12490	–	28.0	20.0	–	–	–	–
	PM-12490	–	26.0	17.0	–	–	–	–
	PM-12490	–	29.0	16.0	–	–	–	–
	PM-12490	–	26.0	17.0	–	–	–	–
	PM-12490	–	28.0	20.0	–	–	–	–

**Notes:**

GL, Greatest length; PB, proximal breadth; PD, proximal depth; DB, distal breadth; DD, distal depth; MB, midshaft breadth; MD, midshaft depth. Villisca (this table). Bat Cave (Hawksley, Reynolds & Foley, 1973, Table 2). Brynjulfson caves no. 1 and 2 (Parmalee & Oesch, 1972, Table 6). Carroll Cave (Hawksley, Reynolds & McGowan, 1963, Table 3; Hawksley, Reynolds & Foley, 1973). Friesenhahn Cave (Graham, 1976, Table 12). Marlow (Cifelli, Smith & Grady, 2002, 94). Powder Mill Creek Cave (Galbreath, 1964, 233). Zoo Cave (Hood & Hawksley, 1975, Table 3). Rancho La Brea (this table).

1 PB and PD are switched in the original publication.

2 Complete radius original cat no. UNSM 9251.

3 Average of two specimens in the collections at the Texas Memorial Museum.

**Figure 7:**
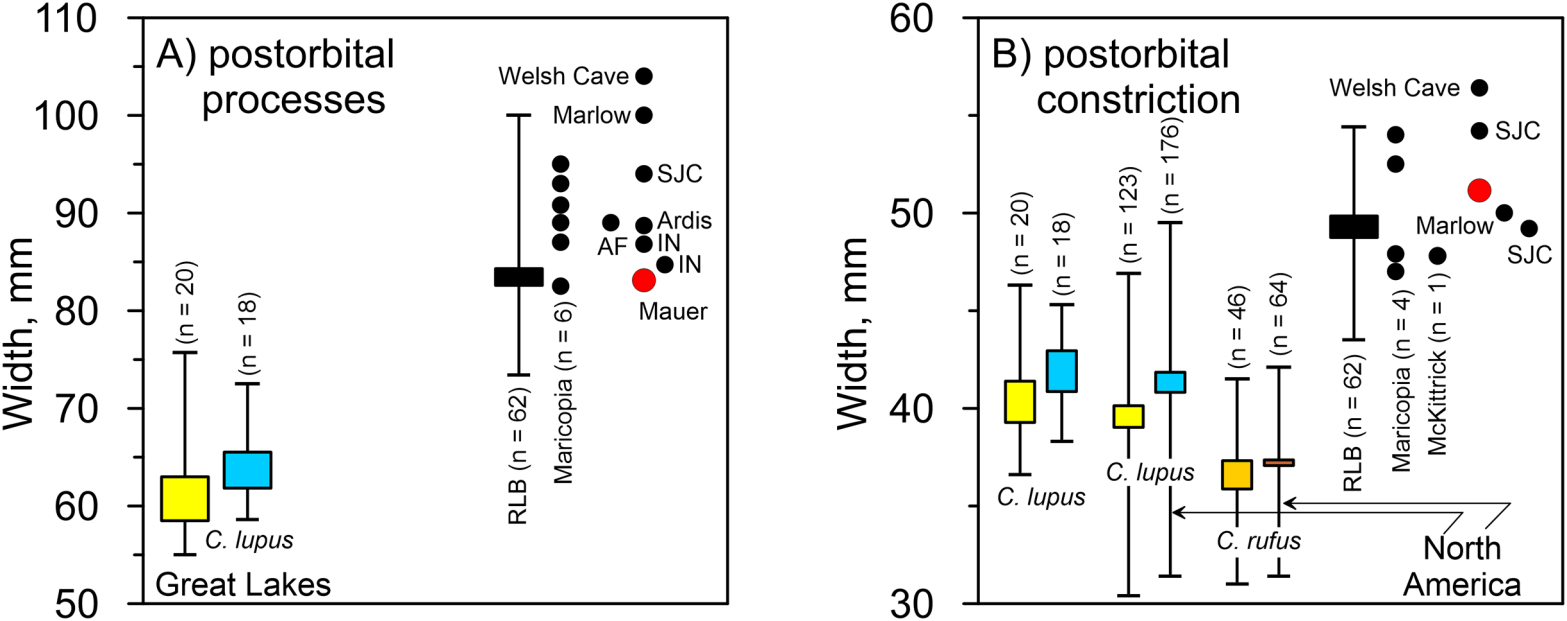
Univariate arrays of cranial measurements on *Canis lupus*, *Canis rufus*, and *Aenocyon dirus*. (A) Width across postorbital processes. (B) Width across postorbital constriction. Results for larger samples are displayed as 95% confidence interval box plots with range whiskers. *A. dirus* uses black symbology, except for Mauer (red). Paired yellow/blue and light brown/dark brown boxes are females and males, respectively. Labeling is minimized to reduce clutter. AF, American Falls. IN, Ingleside. RLB, Rancho La Brea. SJC, San Josecito Cave. Table 3. Table S1. Data S1. Data S2. Data S5. Illustration credit: Matthew G. Hill.

*Mauer cranium*. Direct comparison with the female Alaskan *C. lupus* cranium and indirect, online comparison with a three-dimensional scan of a *C. rufus* cranium indicates the temporal ridges, sagittal crest, and zygomatic remnants in the Mauer specimen are far more robust than in the comparative taxa. The external occipital protuberance, of which only the base is preserved, was apparently massive and probably extended well beyond the occipital cliff. The basioccipital is also exceptionally broad with massive tympanic bullae. The occipital region closely matches that described in *A. dirus* at RLB (Merriam, 1912, 226). In posterior view, the lateral occipital ridges converge dorsally to form a relatively sharp, compressed lambdoidal crest. In *C. lupus*, the lateral ridges gradually converge to form a relatively round, broad lambdoidal crest (Fig. 4). Finally, standard width measurements across the postorbital processes and at the postorbital constriction (Table 3) fall within the ranges in *A. dirus* (Figs. 7A, 7B). In these attributes, there is minimal overlap with *C. lupus* and no overlap with *C. rufus*. These findings are the basis for assigning the Mauer cranium to *A. dirus*.

**Table 3:** Measurements on the Mauer *Aenocyon dirus* cranium.

No.	Attribute (von den Driesch, 1976)	mm
4	Length of basicranial axis	49.6
22	Diameter of auditory bulla	31.1
23	Width across mastoid processes	93.9
24	Width across external auditory meatus	83.5
25	Width across occipital condyles	53.1
27	Width of foramen magnum	27.0
28	Height of foramen magnum	16.9
29	Width of neurocranium	83.9
31	Width across postorbital constriction	51.2
32	Width across postorbital processes	83.1
39	Occipital height without sagittal crest	65.8

**Figure 8:**
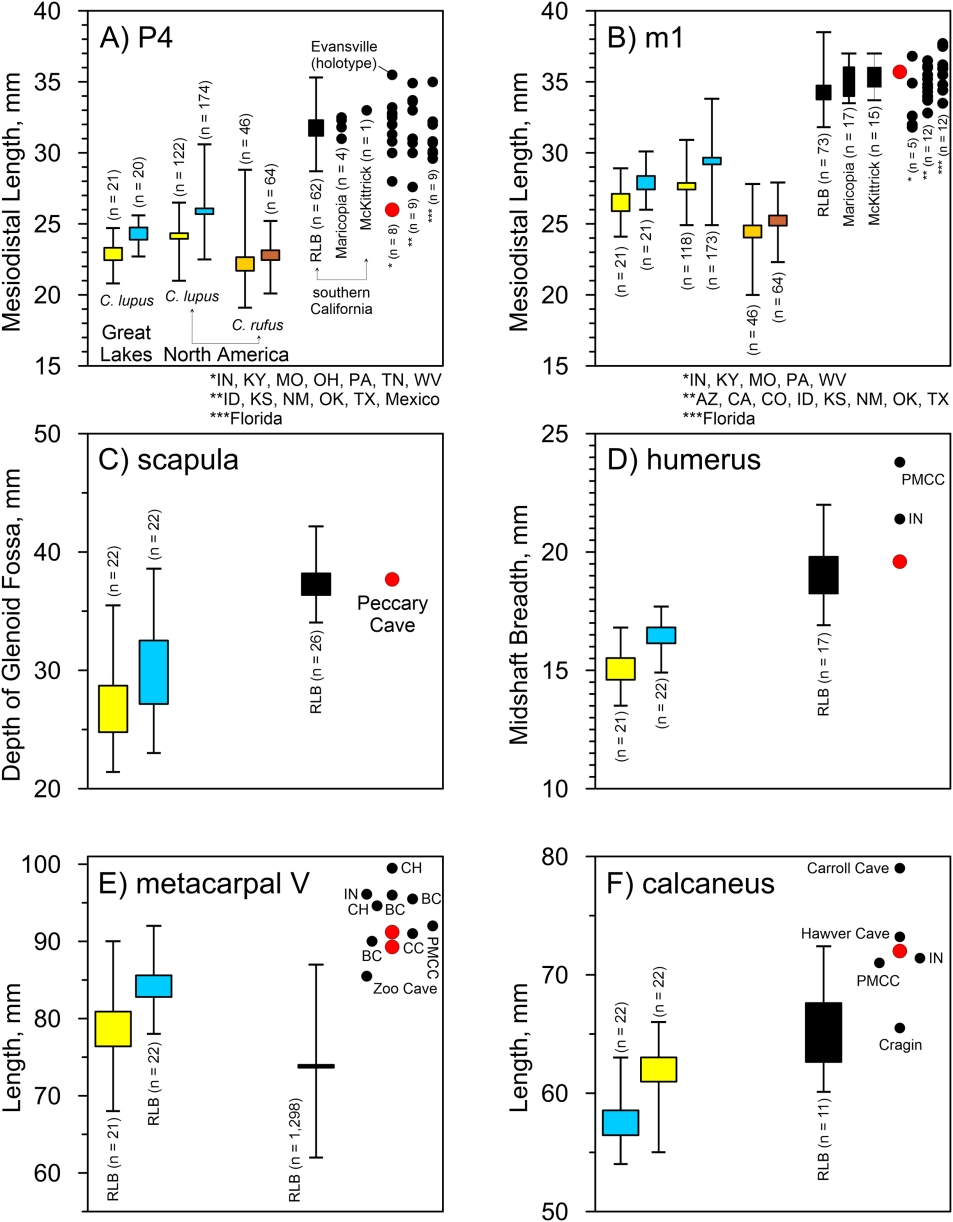
Univariate arrays of selected measurements on *Canis lupus*, *Canis rufus*, and *Aenocyon dirus*. (A) P4 mesiodistal length. (B) m1 mesiodistal length. (C) Scapula, depth of glenoid fossa. (D) Humerus midshaft breadth. (E) Metacarpal V length. (F) Calcaneus length. Results for larger samples are displayed as 95% confidence interval box plots with range whiskers. *A. dirus* uses black symbology, except for Peccary Cave (red). Paired yellow/blue and light brown/dark brown boxes are females and males, respectively. Labeling is minimized to reduce clutter. BC, Bat Cave. CC, Carroll Cave. CH, Cherokee Cave. IN, Ingleside. PMCC, Powder Mill Creek Cave. Tables 4, 5. Table S1. Data S1–S5. Illustration credit: Matthew G. Hill.

*Peccary Cave*. Morphological diagnoses rely on metric comparisons of several specimens. There are two P4s: a complete tooth (UA-4362) and a mesiolingual enamel fragment (UA-4368). The complete tooth is small compared to *A. dirus*, and in fact, it approximates the size of the tooth in *C. lupus* (Fig. 8A). There is no overlap in mesiodistal length with *C. rufus*, except for one female from southeast Missouri (UNSM 244528). However, the tooth displays a reduced deuterocone, as does the enamel fragment, which is consistent with *A. dirus* (Merriam, 1912, 228). Only the talonid portion of the M1 (UA-4374) is present, and notably as in *A. dirus*, the hypocone ridge does not reach the anterior side of the protocone (Merriam, 1912, 231). Of the four m1s, three are fragments (UA-4363, -4364, -4365) and one is a complete tooth (UA-4378) covered in a thin coating of CaCO3. Except for the overall robusticity of the fragments and the mesiodistal length of the complete specimen, they do not retain or expose features to distinguish them from *C. lupus*. Although the length of the complete specimen (35.7 mm) (UA-4378) is perhaps a millimeter or two greater than its actual length because of CaCO_3_ encrustation, it is larger than those in *C. lupus* and *C. rufus* and falls within the *A. dirus* size range (Fig. 8B). The buccolingual breadth of the tooth is too encrusted in CaCO3 for reliable measurement.

**Table 4:** Measurements on *Aenocyon dirus* metacarpi V, and two metacarpi III from Peccary Cave.

		Attribute, mm					
Record	Cat. no.	GL	PB	PD	DAB	DAD	MB
Peccary Cave, Arkansas	UA-2958	89.3	18.3	14.6	12.4	–	12.6
Peccary Cave, Arkansas	UA-2959	–	18.2	–	–	–	–
Peccary Cave, Arkansas	UA-2960	91.2	21.2	17.2	15.9	16.1	12.9
Peccary Cave, Arkansas	UA-2962 (MCIII)	–	12.3	17.4	–	–	–
Peccary Cave, Arkansas	UA-2965 (MCIII)	–	13.2	18.5	–	–	–
Bat Cave, Missouri	–	90.0	–	–	–	–	–
Bat Cave, Missouri	–	96.0	–	–	–	–	–
Bat Cave, Missouri	–	95.5	–	–	–	–	–
Carroll Cave, Missouri	CMS-14	91.0	–	–	–	–	–
Cherokee Cave, Missouri	AMNH-45732	99.5	–	–	–	–	14.5
Cherokee Cave, Missouri	AMNH-45732	94.6	–	–	–	–	13.6
PMCC, Missouri	P-249 (right)	92.0	17.3	18.9	–	14.5	–
Zoo Cave, Missouri	CMSU-573.22	85.5	–	–	–	–	–
Ingleside, Texas	TMM-30967	96.1	12.1	19.1	–	15.0	–

**Note:**

GL, Greatest length; PB, proximal breadth; PD, proximal depth; DAB, distal articular breadth; DAD, distal articular depth; MB, midshaft breadth. Peccary Cave (this table). Bat Cave and Carroll Cave (Hawksley, Reynolds & McGowan, 1963, Table 3). Cherokee Cave (Simpson, 1949, Table 2). Ingleside (Lundelius, 1972, Table 3). Powder Mill Creek Cave (PMCC) (Galbreath, 1964, Table 2). Zoo Cave (Hood & Hawksley, 1975, Table 3).

**Table 5:** Measurements on *Aenocyon dirus* calcanea.

		Attribute, mm			
Record	Cat. no.	GL	GB	GD	SL
Peccary Cave, Arkansas	UA-2964	72.0	30.0	34.0	52.2
Carroll Cave, Missouri	CMS-14	79.0	–	–	–
Powder Mill Creek Cave, Missouri^1^	P-249	71.0	30.0	32.0	–
Cragin Quarry (KU locality 7), Kansas	KU-4613	65.5	30.0	–	–
Ingleside, Texas	TM-30967	71.4	26.3	32.9	–
Hawver Cave, California	21475	73.2	27.0	–	–
Rancho La Brea, California	PM-12490	63.6	24.1	–	42.6
	PM-12490	69.4	26.0	–	49.4
	–	67.4	24.3	–	48.4
	PM-12490	67.4	25.3	–	48.5
	PM-12490	62.2	23.8	–	44.9
	PM-12490	64.4	23.6	–	47.7
	PM-12490	62.1	24.5	–	44.3
	PM-12490	62.0	22.1	–	43.3
	PM-12490	72.4	28.0	–	51.4
	PM-12490	65.4	25.0	–	47.8
	PM-12490	60.1	22.8	–	44.4

**Notes:**

GL, Greatest length; GB, greatest breadth; GD, greatest depth; SL, shaft length. Peccary Cave (this table). Carroll Cave (Hawksley, Reynolds & McGowan, 1963, Table 3). Hawver Cave (Stock, 1918, 479). Ingleside (Lundelius, 1972, Table 3). Powder Mill Creek Cave (Galbreath, 1964, 234). Cragin Quarry (Schultz, 1969, 53). Rancho La Brea (this table).

1 Galbreath, 1964, 241) misreports the greatest length is 69 mm.

Several postcranial specimens offer useful diagnostic information. The only standard measurement obtainable on the scapula (UA-2805) is the depth of the glenoid fossa. It minimally measures 37.7 mm due to minor damage at both measurement points. Still, the feature is larger than in *C. lupus* and in line with *A. dirus* (Fig. 8C). The same size relationships occur in the midshaft breadth of the humerus (19.6 mm) (UA-1132) (Fig. 8D). Two metacarpi V (UA-2958, UA-2960) are similar in length to those from several Missouri caves and the longest specimens from RLB (Table 4). They are about a centimeter shorter than one from Cherokee Cave (Fig. 8E). Moreover, the calcaneus (UA-2964) is about the same length as those from Hawver Cave, Ingleside, Powder Mill Creek Cave, and the largest ones from RLB, all of which are substantially shorter than the Carroll Cave specimen (Table 5) (Fig. 8F). The element is typically smaller in *C. lupus*.

The weight of metric and nonmetric observations on the Peccary Cave large canid remains is consistent with *A. dirus*. This diagnosis is extended to the remainder of the sample.

### ZooMS diagnoses

**Figure 9:**
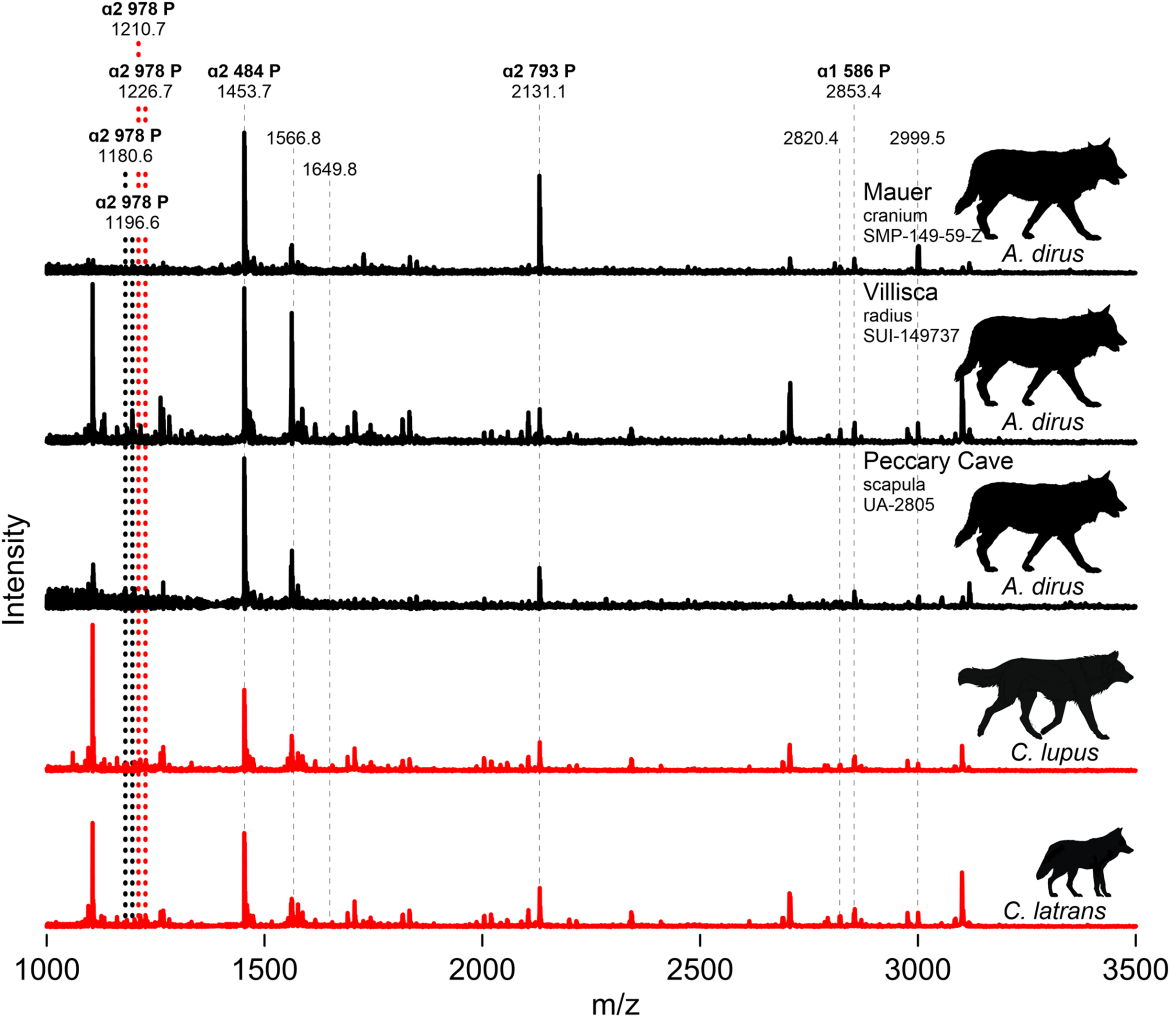
MALDI-TOF spectra. Marker peptides are indicated by dashed lines. Data S6. Illustration credit: Matthew G. Hill.

We attempted to extract collagen from both Iowa specimens, along with the three directly dated specimens (UA-2964, -2958, -2959), the humerus (UA-1132), and the scapula (UA-2805) from Peccary Cave for ZooMS. Only the Iowa specimens and the Peccary Cave scapula produced usable substance. Figure 9 profiles partial MALDI-TOF MS spectra of the tryptic digests of bone collagen from these specimens and modern *C. lupus* and *C. latrans* (Data S6). The paleontological samples are identified as canid based on the presence of the ɑ2 484 peptide at 1453.7 m/z, the ɑ2 793 peptide at 2,131.1 m/z, and the ɑ1 586 peptide at 2,853.4 m/z. The modern canids are well characterized (Buckley et al., 2009) and have ɑ2 978 peptide peaks at 1,210.7/1,226.7 m/z. The Villisca, Mauer, and Peccary Cave spectra are indistinguishable from each other and importantly, do not exhibit the ɑ2 978 peak of *C. lupus and C. latrans*. In *A. dirus*, that peptide appears to have shifted to 1,180.6/1,196.6. A difference in the ɑ2 978 peptide is also suggested by the amino acid sequence published by Perri et al. (2021) for *A. dirus*.

ZooMS thus indicates the Mauer cranium, Villisca radius, and Peccary Cave scapula are not *Canis*. This finding excludes the possibility of *C. lupus*, which occurs at Natural Trap Cave, Wyoming (Meachen, Brannick & Fry, 2016), and *C. rufus*, which occurs in the regional paleozoological record (Nowak, 2003, Fig. 9.7). The only viable alternative taxonomic candidate for them is *A. dirus*, which is also assigned to the remainder of the Peccary Cave large canid remains.

### Radiometric and isotopic results

AMS radiocarbon dates, isotopic results, and related data for both Iowa specimens and three Peccary Cave specimens are provided in Table 6. The C:N ratios (3.1–3.1) suggest the measured samples are not severely degraded or affected by exogenous contaminants, indicating that the dates and isotopic results are likely reliable (Ambrose, 1990, 447; Talamo et al., 2021, 63-64; van Klinken, 1999, 691). All of the dates fall within MIS 2 (29,000–11,700 cal B.P.). Mauer dates to 29,040–28,410 cal B.P., Peccary Cave to 25,350–21,405 cal B.P., and Villisca to 14,325–14,075 cal B.P.

**Table 6:** AMS radiometric and isotopic results for Iowa and Peccary Cave *Aenocyon dirus*.

Location	Cat. no.	Lab no.	Age ^14^C yr B.P. (±1σ)	Calibrated age ^14^C yr B.P. (2σ range)	δ^15^N‰ (AIR)	δ^13^C‰ (VPBD)	N%	C%	C:N (atomic)
Mauer, Iowa	SMP-149-59-Z	Aeon-1381	24,460 ± 110	29,040–28,410	7.5	−18.9	15.2	43.0	3.3
Villisca, Iowa	SUI-149737	UCIAMS-223273	12,270 ± 30	14,325–14,075	5.8	−19.8	15.8	41.8	3.1
Peccary Cave, Arkansas	UA-2964	UCIAMS-165393	17,880 ± 100	22,030–21,405	6.1	−18.4	14.9	40.8	3.2
Peccary Cave, Arkansas	UA-2958	UCIAMS-175965	19,910 ± 70	24,155–23,785	7.4	−18.9	13.9	38.7	3.2
Peccary Cave, Arkansas	UA-2959	UCIAMS-175966	20,850 ± 80	25,350–24,910	8.0	−18.4	14.9	40.9	3.2

## Discussion

### Occurrences

Taking into consideration minor changes to Dundas’ (1999) inventory, 26 records published after his census, the addition of 13 records he overlooked or omitted from his study, and the two new Iowa occurrences, there are now 166 *A. dirus* records in southern North America (Fig. 1) (Data S7). Roughly half (*n* = 70) of these records—including Mauer and Villisca—are not reported in the Neotoma Paleoecology Database.

Notable additions and updates to the *A. dirus* record include:

1. A nearly complete cranium and several other elements, including measurements, from Burnham, Oklahoma (Czaplewski, 2003).
2. An update on the partial skeleton from Marlow, Oklahoma (Cifelli, Smith & Grady, 2002).
3. Notice of a complete skeleton from Megenity Peccary Cave, Indiana, belonging to an adult male that had fallen into a 6-m steep-walled pit and became trapped (Richards, 1995, 90; Richards & Whitaker, 1997, 149).
4. Measurements on the complete skull (cranium + mandibles) from Ardis, South Carolina (Sanders, 2002, Table 3).
5. Descriptions of material from Medicine Hat (Surprise Bluff), Alberta (Reynolds et al., 2023), Kincaid Shelter, Texas (Johnson & Moretti, 2022), Natural Trap Cave, Wyoming (Meachen, Brannick & Fry, 2016; Redman et al., 2023, 44), Murray Springs and Lehner Ranch, Arizona (Hemmings, 2007, 103; Saunders, Baryshnikov & Seymour, forthcoming), and Peccary Cave, reported here.
6. The first records for Iowa, reported here, and for Nevada, from Tule Springs (Scott & Springer, 2016).
7. The addition of the taxon to faunal lists for Zesch Cave, Texas (Sagebiel, 2010), and Ladds, Georgia (Nowak, 2002, 107).
8. Another specimen, an m1, from Stratum 2/V at Vero, Florida (Adovasio, Hemmings & Vento, 2024, 83; Hemmings et al., 2018, 87).
9. Direct AMS dates from Natural Trap Cave, Wyoming, and Guy Wilson Cave, Tennessee (Perri et al., 2021).
10. Development of a series of ≥76 direct AMS dates for RLB (Friscia et al., 2008; Fuller et al., 2014; Fuller et al., 2015; Fuller et al., 2020; O’Keefe et al., 2023).
11. Age-bracketing of the material from Hall’s Cave, Texas (Seersholm et al., 2020; Waters et al., 2021, 9).
12. Reassignment of *C. mississippiensis* to *C. lupus*, with the provenance being the lead region, and verification of the Blue Mounds, Wisconsin *C. lupus* record (Addendum).
13. Placement of the provenance of a mandible reported by Merriam (1912, 221–222, 242–243) and Hibbard (1939, Table 1) at Sheridan, a ghost town in Logan County, Kansas (Addendum).

**Figure 10:**
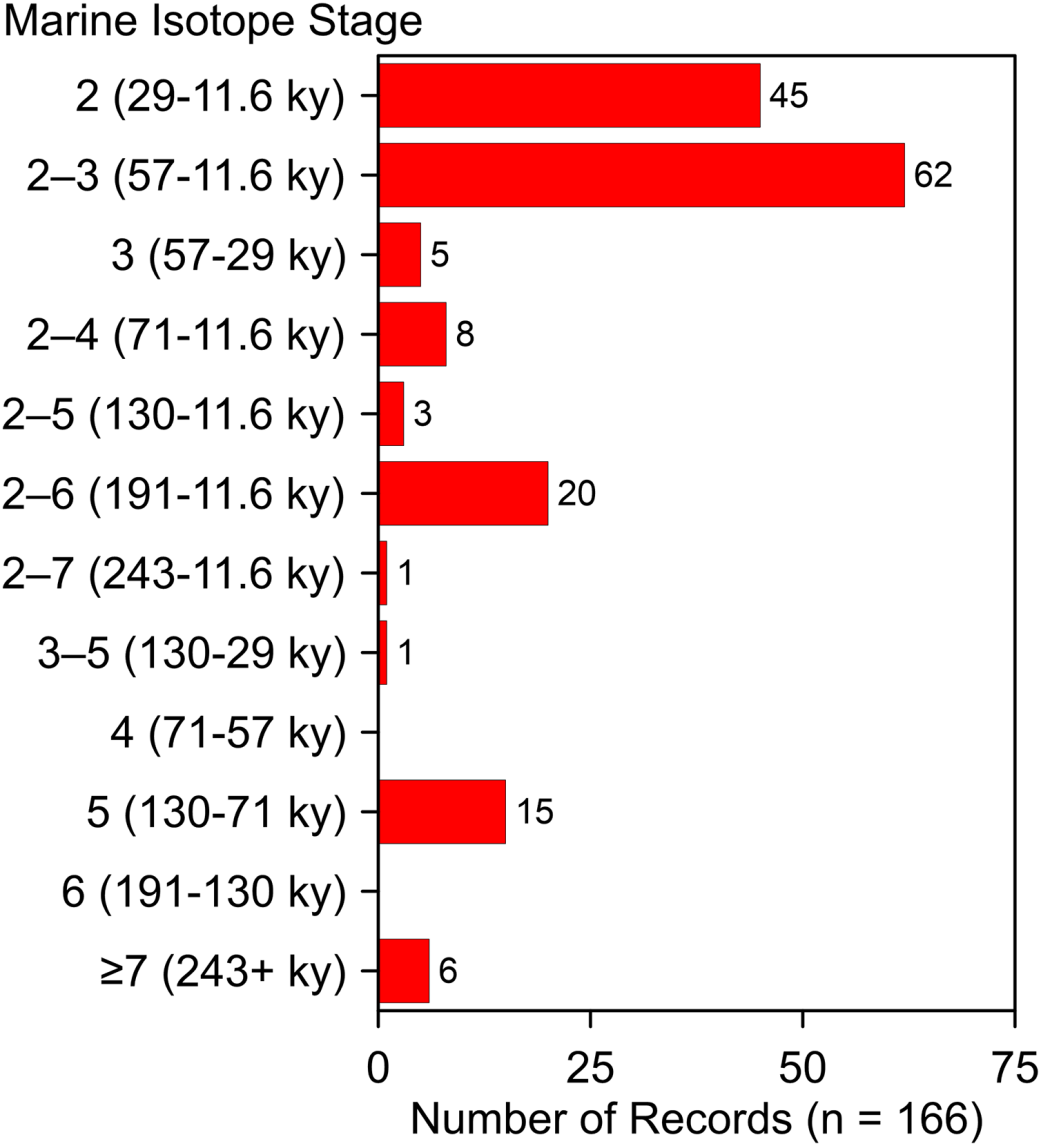
Age-frequency distribution of *Aenocyon dirus* records in southern North America. MIS age boundaries (Lisiecki & Raymo, 2005). The terminus of the Younger Dryas is regarded as the end of MIS 2. Rancho La Brea is tallied one time in the MIS 2–3 bin. Data S7. Illustration credit: Matthew G. Hill.

These additions and updates do not substantially change the general character of the record reported by Dundas (1999). The species ranges in time from as early as MIS 19 at Fairmead Landfill and Irvingtonian 2, California (Tedford, Wang & Taylor, 2009, 132; Trayler et al., 2015, 132) to MIS 2. Two-thirds (*n* = 112) of the occurrences date to MIS 2, MIS 2–3, and MIS 3 (Fig. 10). Geographically, it spans from the Pacific Coast to the Atlantic Coast, and from El Cedral, Mexico, in the south to Medicine Hat, Alberta, in the north (Fig. 1). Actual absence, non-preservation, and inadequate sampling account for the spotty distribution of occurrences in some regions (Grayson, 1981). Notable clusters of occurrences are found in Florida (*n* = 32), California (*n* = 29), and in and around the Ozarks (*n* = 13). The southern Great Plains also contains a relatively large number of occurrences. The Ozark record represents the largest concentration of *A. dirus* material in the interior of southern North America, encompassing a variety of find-types, from a few bones or teeth to nearly complete skeletons. State-line records from locations such as Natural Trap Cave in northern Wyoming, and Cherokee Cave and Herculaneum Fissure in Missouri, strongly suggest the taxon ranged into Montana and Illinois, for example (Merriam, 1912, 222).

### Biogeography

The distribution of occurrences highlights generalist adaptations in *A. dirus*. The taxon apparently thrived in various environments with different prey options. For instance, in California over the past 750,000 years (MIS 2–19), the combination of stable isotope data and faunal associations suggest the taxon preyed mainly on *Camelops*, *Bison*, *Equus*, and *Hemiauchenia* in warm, xeric settings such as open prairies, mixed woodlands, and scrublands (DeSantis et al., 2019; Fuller et al., 2014; Fuller et al., 2020; Trayler et al., 2015; O’Keefe et al., 2023). Similarly, at Cutler Hammock, Florida (late MIS 2), it lived a warm environment transitioning from relatively closed mesic conditions to open xeric conditions. Consequently, its diet shifted from browsers such as *M. fossilis*, *P. compressus*, and *Odocoileus* to grazers such as *Bison*, *Equus*, *Hemiauchenia* (Munson, 2022, 31–32; cf. D’Amo, 2001, 39–43). The Mauer, Villisca, and Peccary Cave records provide information from the interior of North America, within a continental environmental context.

Proxy evidence from Logan Quarry, on the Boyer River (Fig. 1C), about 30 km downstream from the Mauer find-spot, indicates the animal inhabited an environment that was transitioning from full boreal to full glacial conditions (Baker et al., 2009, Table 1). Fifteen thousand years later, during the early Bølling-Allerød Chronozone (14,640–12,850 cal B.P.) (Byun et al., 2021; Gonzales & Grimm, 2009, 242), the Villisca animal inhabited a boreal parkland (Rhodes & Semken, 1986, 109).

**Figure 11:**
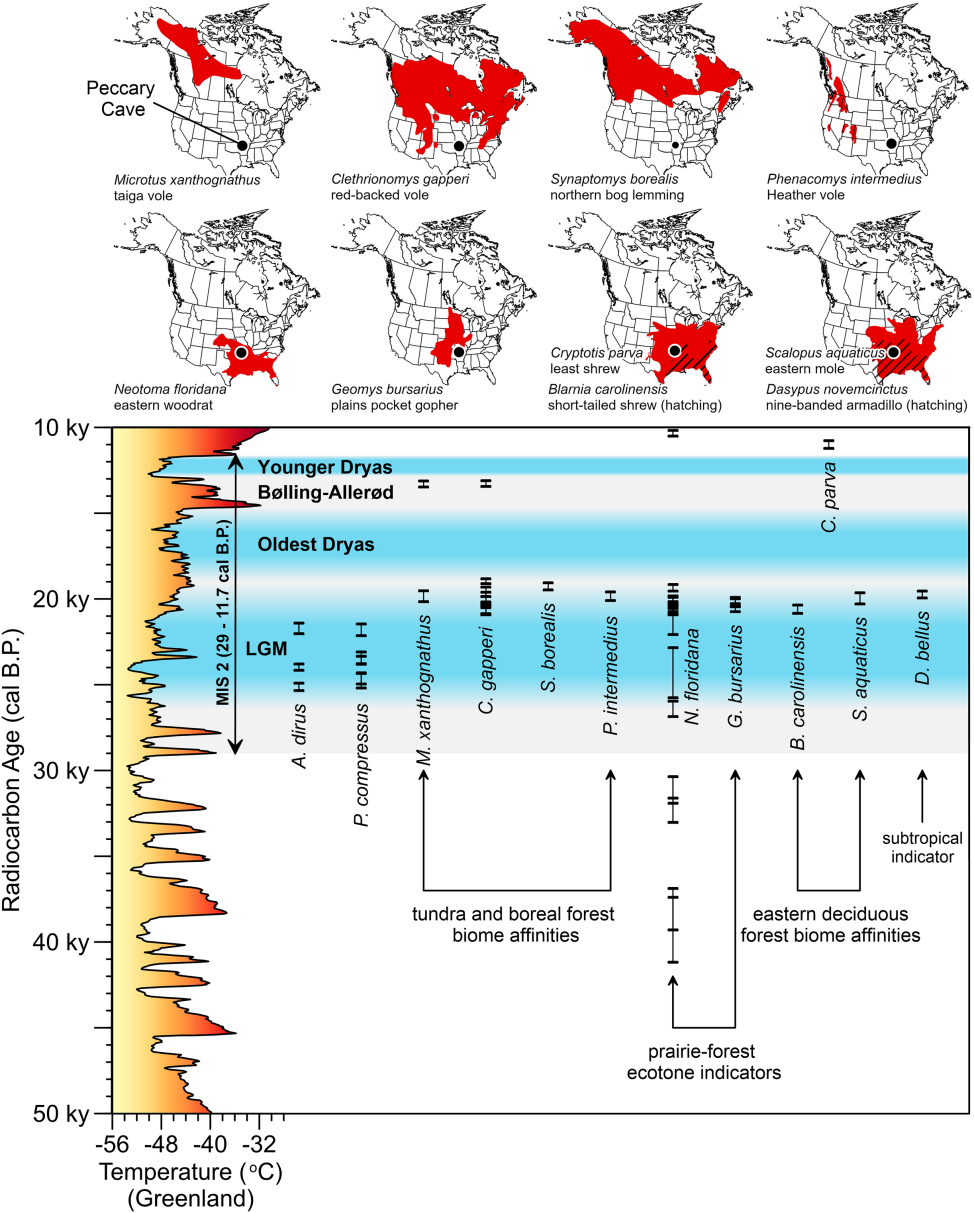
AMS radiocarbon dates for Peccary Cave and modern ranges of selected small mammals. The vertical bars capture the two-sigma range of each assay. In two instances, two small mammal taxa are displayed on the same range map. Marine Isotope Stage 2 (MIS 2) is shaded gray. Dates for the Last Glacial Maximum (LGM) (Northern Hemisphere), Oldest Dryas, Bølling-Allerød, and Younger Dryas Chronozones are for the midcontinent (Carlson & Winsor, 2012, 3; Clark et al., 2009; Gonzales & Grimm, 2009, 241–242). The terminus of the Younger Dryas is regarded as the end of MIS 2. Reconstructed temperature for Greenland over the last 50,000 years (Alley, 2000; Alley, 2004) is profiled on the y-axis. *A. dirus* dates (Table 6). *P. compressus* dates (Wilson & Hill, 2020, Table 1). Small mammal dates (Data S8). Shapefiles for small mammal ranges downloaded from Map of Life (www.mol.org). Illustration credit: Matthew G. Hill.

The environment *A. dirus* inhabited is clearer at Peccary Cave. During the LGM, the Ozarks were covered in a *Picea*- and *Pinus*-dominated boreal forest interspersed with some deciduous hardwoods that has no modern analog (Jackson et al., 2000; King, 1973). The understory and forest openings were vegetated with *Cyperaceae*, *Poaceae*, and *Artemisia* shrubs and forbs, among other taxa. Palynological evidence from Cupola Pond, 200 km northeast of Peccary Cave (Fig. 2A), indicates this forest persisted until 15,000 cal B.P. (Jones, Williams & Jackson, 2017, Figure 7). Small mammal taxa from Peccary Cave also record a non-analog environment (Semken, Graham & Stafford, 2010; Stafford et al., 1999). As illustrated in Fig. 11, the majority of small mammals date to approximately 20,000 cal B.P. (Data S8), and significantly, the associated range maps demonstrate contemporaneity of several taxa with modern distributions that do not overlap, specifically, northerly tundra/boreal taxa and several southerly prairie/deciduous forest taxa. Compared to the modern situation, mean January and July temperatures were 15–20°C and 10°C colder, respectively, and there was ~40 mm less precipitation (Jackson et al., 2000, 500).

### Diet

Leaving aside subadult and adult proboscideans, accounts of prey selection and hunting in *C. lupus* (Mech, Smith & MacNulty, 2015; Peterson & Ciucci, 2003) reveal that any herbivore co-existing with *A. dirus* was not immune to predation. In western Iowa, an absence of direct dates on potential prey species makes it difficult to put *A. dirus* diet on solid ground at the present time. However, the list of practical candidates is short. It includes *Bootherium*, *Megalonyx*, and *Cervalces*, whose remains are relatively abundant in the regional fossil record (Barbour, 1931; Churcher, 1991; Churcher & Pinsof, 1988; Dulian, 1975; Hay, 1914, 78–79, 306; McDonald, 2012). *P. compressus* is known only from several locations in southwestern Wisconsin, northeast Iowa, and northwestern Illinois (Wilson & Hill, 2020, Figure 1), while its solitary counterpart, *M. fossilis* (Kurtén & Anderson, 1980, 296), has yet to be reported in the state. Months-old proboscideans were probably uncommon prey due to the unpredictable nature of the necessary circumstances for attacks to occur (see Van Valkenburgh et al., 2016).

Comparison of stable nitrogen isotope results obtained for *A. dirus* from Villisca and for *S. fatalis* from a sand-and-gravel operation 2 km north of Shenandoah (WGS 84 40.76N, 95.37W) (Fig. 1C) (Hill & Easterla, 2023, Table 1) casts light on the diet of these carnivores during the Bølling-Allerød in southwest Iowa. Briefly, studies on the diet of predators that consume mostly vertebrate prey indicate the difference between the isotopic composition of predator and prey bone collagen directly relates to the isotopic enrichment that attends each trophic step. The δ^15^N value of the collagen, in particular, increases 3–5‰ with each trophic step, from primary consumers to top consumers (Bocherens & Drucker, 2003; Schoeninger & DeNiro, 1984). The *S. fatalis* result (δ^15^N = 8.2‰) (Hill & Easterla, 2023, Table 1) is a trophic step higher than the *A. dirus* result (δ^15^N = 5.8‰) (Table 1). While the absence of complementary isotopic data on potential prey constrains what can be inferred from these values, it tentatively suggests the two taxa were possibly not in regular competition for the same prey and occupied different niche spaces, as appears to be the case at RLB (DeSantis et al., 2019, 2489). This general idea is supported by compelling evidence that *S. fatalis* was a solitary ambush hunter, whereas *A. dirus* hunted in packs (Brown et al., 2017).

In the Ozarks, *P. compressus* was ideal prey, an idea alluded to decades ago by Hood & Hawksley (1975, 14). With adults weighing 50–75 kg (Guilday, Hamilton & McCrady, 1971, 291), it was probably the most common medium-sized herbivore in many late Quaternary faunal communities (Wilson & Hill, 2020). To this point, actualistic work in savanna and temperate ecosystems reveals a high level of fidelity between the number of a taxon’s bones on the landscape and its living abundance (Miller, 2011; Miller, 2012; Western & Behrensmeyer, 2009). If this relationship holds for closed settings such as Peccary Cave, then *P. compressus* (MNI = 100) outnumbered *A. dirus* (MNI = 3) outside the cave by a wide margin, as would be expected given fundamental trophic linkages (Pimm, Lawton & Cohen, 1991). Second, while its defensive weaponry—large canines or ‘tusks’—conferred some protection from predation, *P. compressus* was far less capable of inflicting serious counter-injuries during attacks than other coexisting megaherbivores. Compared to *P. compressus*, these animals weighed hundreds of kilograms and possessed potentially lethal horns, antlers, clawed forelimbs, and powerful, rapid-fire hooves to combat take-down attempts (Brown et al., 2017; Van Valkenburgh & White, 2021).

**Figure 12:**
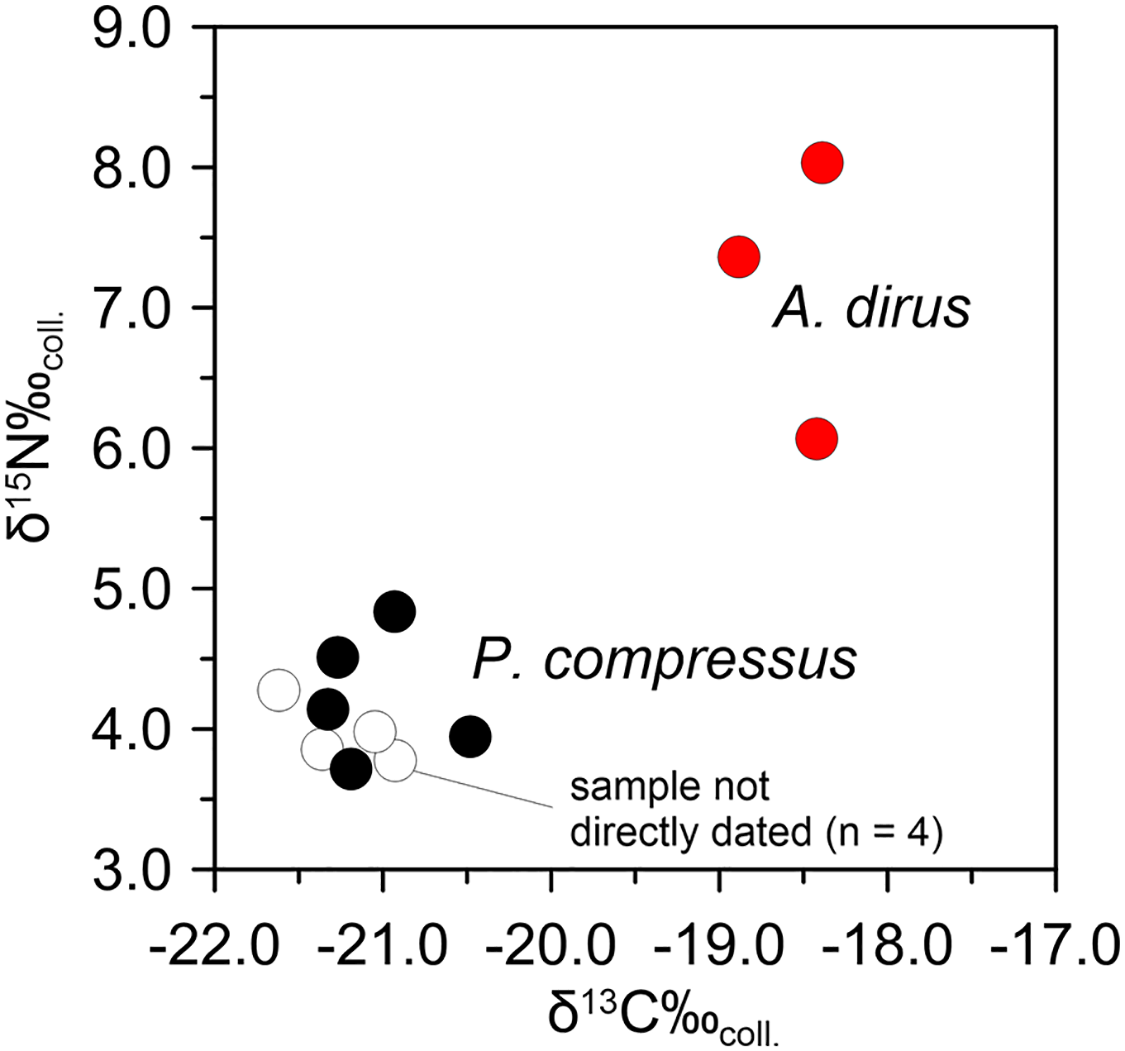
Bivariate array of δ^13^C and δ^15^N values for *Aenocyon dirus* and *Platygonus compressus* from Peccary Cave. Tables 6 and 7. Illustration credit: Matthew G. Hill.

Following the same analytical tack as above, stable isotope analyses on *A. dirus* and *P. compressus* from Peccary Cave support the existence of this inferred predator-prey relationship between 25,000 cal B.P. and 21,000 cal B.P. Isotopic data for *A. dirus* and *P. compressus* are provided in Tables 6 and 7. The expected relationship between results for the taxa holds true (Fig. 12). The δ^15^N_coll_ values for *A. dirus* (range 6.1–8.0‰) are 3–5‰ higher than those for *P. compressus* (range 3.7–4.8‰). While another prey taxon such as *Bootherium* or *Megalonyx* might be driving the *A. dirus* values, *P. compressus* is the most probable candidate behind them in this regional LGM faunal community.

**Table 7:** AMS radiometric and isotopic results on *Platygonus compressus* from Peccary Cave.

Acc. no.	UCIAMS	Age ^14^C yr B.P. (± 1σ)	δ^15^N‰	δ^13^C‰	C:N (atomic)
UA-916^1^	175968	19,600 ± 70	4.1	−21.3	3.2
UA-918^1^	165400	20,610 ± 140	3.7	−21.2	3.2
UA-923^1^	165398	19,460 ± 130	4.5	−21.3	3.2
UA-930^1^	165399	17,990 ± 100	3.9	−20.5	3.3
UA-931^1^	175967	20,500 ± 80	4.8	−20.9	3.2
UA-914^2^	–	–	4.0	−21.0	
UA-915^2^	–	–	4.3	−21.6	
UA-921^2^	–	–	3.9	−21.4	
UA-929^2^	–	–	3.8	−20.9	

**Notes:**

Results on right humeri.

1 Previously published (Wilson & Hill, 2020, Table 1).

2 Assayed at the Stable Isotope Laboratory, Department of the Earth, Atmosphere, and Climate, Iowa State University.

### *Aenocyon dirus* and *Platygonus compressus* use of Peccary Cave

The radiocarbon dates indicate the use of the Peccary Cave by *P. compressus* and *A. dirus* between 25,000 and 21,000 cal B.P. (Fig. 11), which apparently bookends the period when these animals were able to navigate the passageway leading to the main chamber (Fig. 2C). Binford (1981, 200) notes a similar cave-use situation in Alaska involving *C. lupus* and *Ovis dalli*. By 21,000 cal B.P., the passageway was partially choked with poorly sorted alluvium from overbank flooding of Ben’s Branch, so much so that it appears to have effectively blocked these large animals from entering the cave. This opening, or an unknown one, remained accessible to small mammals until 19,000 cal B.P. when all access points were apparently sealed. Small mammals somehow gained entry again between 13,000 and 10,000 cal B.P. until that opening closed. Later, in the short passage off the south wall of the main chamber, a 10 m chimney opened and a 4.26 m tall debris cone beneath it began to build (Fig. 2C) (Ball & Davis, 1993; Davis, 1969, 196). Several middle Holocene radiocarbon dates on *Neotoma floridana* (Semken, Graham & Stafford, 2010, Table 1) may approximate the timing of chimney formation.

The high MNI, presence of fetuses and droppings, and observations of cave use in *Pecari tajacu* (Sowls, 1997, 67, 130–133) indicate regular use of the cave by *P. compressus*, with one individual dying inside the cave approximately every 45 years (Wilson & Hill, 2020, 8). The rate and duration of visitation probably increased during the winter as animals sought protection from the bitter cold. For *A. dirus*, at least three adult animals died inside the cave over a period of four thousand years. Two of these individuals were relatively old. In Stiner’s (1994, Fig. 12.3) nine-cohort eruption-and-wear sequence for canids, the two right, partial m1s are heavily worn. As best as can be determined, one specimen appears to belong to group VII (UA-4364), and the other to group VIII (UA-4363). The remaining m1, identified as a left buccal fragment (UA-4365), cannot be assigned to a group due to the lack of a readable occlusal surface. However, this specimen was fully erupted and appears to have belonged to a younger adult, relative to those represented by the two right m1s. These observations lead us to infer that the cave periodically functioned as a refuge for aged and infirm animals. Most likely, this mode of accumulation also explains the presence of partial skeletons with heavily worn teeth found at places such as Zoo Cave, Missouri (Hood & Hawksley, 1975, 28), Powder Mill Creek Cave, Missouri (Galbreath, 1964, 235), and Marlow, Oklahoma (Cifelli, Smith & Grady, 2002, 94).

Granting some parallels with *C. lupus*, the evidence does not point to its use as an *A. dirus* den. While den type, location, and sociality are thoroughly documented in *C. lupus* (Packard, 2003, 35–51), actualistic research on the bone debris that accumulates in and around *C. lupus* dens to aid in interpreting paleozoological patterns is sparse but revealing. Foremost, prey remains are rare inside dens but can be relatively common on entrance aprons, and second, these assemblages are “dominated by (heavily gnawed) skulls, teeth, feet, and lower limbs for the large and moderate-size mammals (*i.e*., multi-taxic)” (Binford, 1981, 202; Haber, 1977, Appendix 11; Haynes, 1980, 1982; Mech & Packard, 1990; Murie, 1944, 31; Packard, 2003, Figure 2.4; Peterson, 1977, 107; Young, 1964, 101–102). As well, reuse of bedrock overhangs, nooks, and crannies for denning is common in places where these features are *not* common and, consequently, prey-bone accumulations at these places are relatively large and time-averaged (Haber, 1977, 302). Finally, since whelping typically occurs in dens (Fuller, Mech & Cochrane, 2003, 183–184; Peterson, 1977, 112; see also Stiner, 1994, 286–287, 316–319), sometimes they contain puppy remains (Milideo, 2015, 39).

None of these expectations are met at Peccary Cave. Neonatal or very young *A. dirus* remains are not present. Differential preservation cannot account for this absence since fetal *P. compressus* bones and teeth are relatively common. Second, unambiguous signs of carnivore scavenging of *P. compressus* carcasses in the form of tooth scores, punctures, gastric etching, and destruction of articular ends of bones are limited to four specimens. Also, while the assemblage contains remains from other potentially viable prey species (*i.e*., multi-taxic), specifically, *Cervalces* (*n* = 3), *Bootherium* (*n* = 12), and *Tapirus* (*n* = 1), the representative specimens are isolated teeth. How these remains entered the main chamber is not clear, but it is notable that the *Tapirus* specimen is water-worn, as is a hand-sized chunk of proboscidean long bone. Several other proboscidean remains reported by Quinn (1972, 92) have not been relocated. (The *Equus* tooth mentioned by Quinn (1972, 92) is, as he suggests, modern in age.) Finally, the Ozarks contain 8,500 reported caves (Tennyson et al., 2017, 663) and probably many times that number of small crevices, fissures, and low overhangs. Given this, and at least over the short-term, *A. dirus* inhabiting the Ozarks probably did not often reuse natal dens, which when considered with the fact they are typically used only in the spring for several weeks in Minnesota (Fuller, 1989, Table 1; Mech, 2000, 78) and eight weeks in Alaska (Mech et al., 1998, 103), will make it exceedingly difficult to identify these places from faunal evidence (cf. Emslie & Morgan, 1995; Nye, 2007).

### Sex and body proportions

The partial *A. dirus* skeleton from Powder Mill Creek Cave, Missouri, is inferred to belong to an adult female (Galbreath, 1964, 235), and similarly, the one from Zoo Cave is also mature and “quite small by Missouri standards” (Hood & Hawksley, 1975, 28). The partial skeleton from Marlow, Oklahoma, is also from a small, skeletally and dentally mature individual (Cifelli, Smith & Grady, 2002, 94). If the radii in these individuals approximate the size of adult females, then it seems probable the specimens from Brynjulfson Cave no. 1, Missouri, and the larger of the two from Bat Cave, Missouri, represent adult males (Fig. 6A). These specimens are also longer than in the “large male” (Galbreath, 1964, 241) from Carroll Cave, Missouri. All other things equal, it appears the Villisca radius belongs to a male. The greatest length is closer to that recorded for inferred males than inferred females. It is slightly longer than the longest male radius in the *C. lupus* sample. In prime physical condition, the Villisca animal may have approached 70 kg (Anyonge & Roman, 2006, 211), or about the same as exceptionally large *C. lupus* males (~80 kg) (Mech, 1970, 11–12). (In comparison, the measured live weight of the four heaviest males in the Great Lakes *C. lupus* sample is 44.4 kg.) This estimate aligns with observations that, overall, the morphotype found east of the Rocky Mountains was taller and more robust than its counterpart found west of the Rocky Mountains, and in turn, supports the inference that it was possibly faster and more agile (Kurtén, 1984, 226).

### Timing of extinction

**Figure 13:**
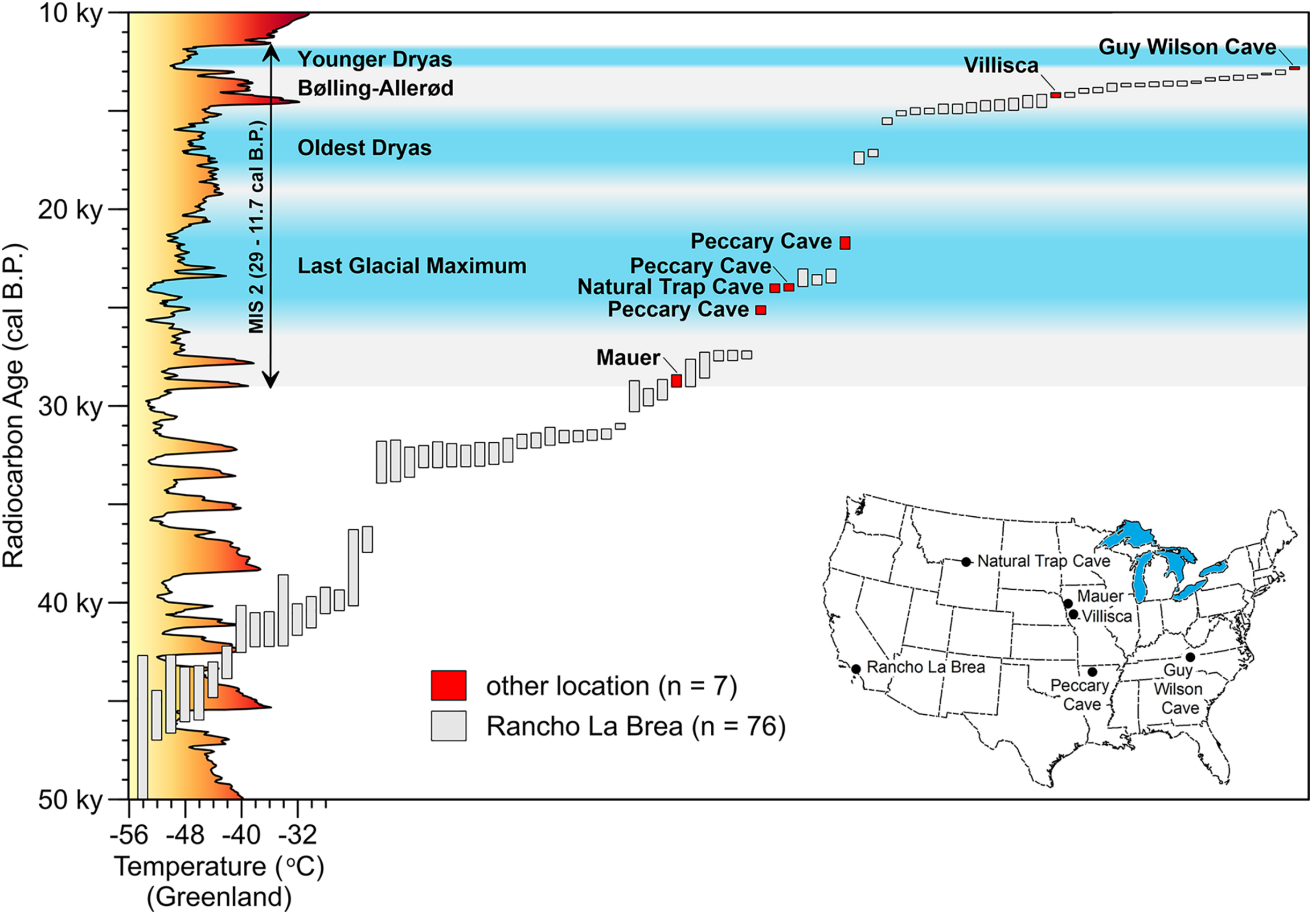
Time-series of AMS radiocarbon dates on *Aenocyon dirus* in southern North America. Vertical bars capture the two-sigma range of each assay. MIS 2 is shaded gray. Dates for the Last Glacial Maximum (Northern Hemisphere), Oldest Dryas, Bølling-Allerød, and Younger Dryas Chronozones are for the midcontinent (Carlson & Winsor, 2012, 3; Clark et al., 2009; Gonzales & Grimm, 2009, 241–242). (The terminus of the Younger Dryas is regarded as the end of MIS 2). Reconstructed temperature for Greenland over the last 50,000 years (Alley, 2000; Alley, 2004) is profiled on the y-axis. Data S9. Illustration credit: Matthew G. Hill.

Outside of RLB, seven direct AMS radiocarbon dates from five localities are available for *A. dirus*. Figure 13 profiles these results in relation to those from RLB (*n* = 76) (Data S9). The RLB series minimally spans the period 50,000–13,000 cal B.P. (MIS 2 and 3), whereas the other dates minimally span the period 29,000–13,000 cal B.P. (MIS 2). Small sample size, dating failures (Cifelli, Smith & Grady, 2002, 94; MacFadden et al., 2012, 708; Perri et al., 2021), and non-preservation of directly dateable material in places such as Florida (Hulbert, Morgan & Kerner, 2009, 546; Morgan, 2002, 21) account for this discrepancy. Biostratigraphic ages of ~42,000 cal B.P. (MIS 3) from Medicine Hat (Surprise Bluff), Alberta (Reynolds et al., 2023, 940) and ~40,000 cal B.P. (MIS 3) from Burnham, Oklahoma (Czaplewski, 2003, 162; Wyckoff & Carter, 2003), for example, are good backfill, however.

The earliest date east of the Rocky Mountains is on the Mauer cranium, followed by three LGM dates from Peccary Cave (Table 6) and one from Natural Trap Cave, Wyoming (24,235–23,795 cal B.P.) (Perri et al., 2021), and then the Villisca date. The average of two assays on a tooth root from Guy Wilson Cave (12,965–12,755 cal B.P.) (Perri et al., 2021) is the current last appearance date for the taxon. Sixteen results from RLB fall between those from Villisca and Guy Wilson Cave, Tennessee. The tightly bracketed *A. dirus* remains from Hall’s Cave, Texas (14,700–12,900 cal B.P.) (Waters et al., 2021, 9) fall between the Guy Wilson Cave and Villisca dates. These observations indicate that *A. dirus* survived in geographically distant locations across southern North America until terminal extinction sometime after 12,800 cal B.P.

## Conclusion

This study documents the first records of *A. dirus* in Iowa and removes the Peccary Cave sample from the list of widely known but minimally published paleozoological evidence. ZooMS was used to verify several morphological diagnoses. The future of this quick, inexpensive, and minimally destructive taxonomic aid is promising.

The *A. dirus* record comprises 166 occurrences across the southern North America, dating from MIS 2 to MIS 19, with notable concentrations in California, Florida, and the Ozark region, and to a lesser extent, on the southern Great Plains. The spotty nature of the record is likely due to inadequate sampling and nonpreservation of fossils, rather than an actual absence of presence in most areas. For instance, there are no obvious reasons that would have kept *A. dirus* from ranging into southern Minnesota, southern Wisconsin, and Illinois, among other places. Based on current understanding, terminal extinction occurred sometime after 12,800 cal B.P. and was relatively synchronous across the region.

The radiocarbon results firmly place *A. dirus* on the eastern Great Plains during the pre-LGM (at Mauer), LGM (at Peccary Cave), and Bølling-Allerød (at Villisca). Main prey species in Iowa are currently speculative, however, at least during the Bølling-Allerød, it appears that *A. dirus* and *S. fatalis* did not regularly compete for the same prey and occupied different niche spaces. Considerations of isotopic data, prey abundance, and prey size suggest *P. compressus* was the main prey to the south in the Ozarks. Additional isotopic data on other taxa and on *A. dirus* from other localities in the Ozarks might also provide clues about variation in preferred hunting habitats, the number of individuals in hunting packs, and prey switching. For example, and at least in theory, *P. compressus* could be grappled by one individual, while animals such as *Bootherium* likely required the cooperation of several individuals. Put differently, regional variation in diet may inform *A. dirus* sociality in a boreal context, and in this way, may complement research in places like southern California (*e.g*., Carbone et al., 2009; Van Valkenburgh et al., 2009).

Currently, radiometric and isotopic data on *A. dirus* is sparse east of the Rocky Mountains. To detect range shifts due to movements of the Laurentide ice sheet, geographic variation in trophic relationships, and spatiotemporal patterns of terminal extinction, it will be necessary to assay more specimens across the region. Similarly, reexamination of *A. dirus* material from caves and other closed settings, focusing on mode of accumulation, is needed to better understand the taxon’s use of these places. In some cases this is relatively clear, as evidenced by deadly accidents at Megenity Peccary Cave, Natural Trap Cave, and RLB. However, in other cases, it remains quite uncertain.

## Addendum

### Reassignment of Joel Allen’s *Canis mississippiensis* to *Canis lupus*

In 1876, Joel Allen provisionally named *Canis mississippiensis* as an extinct species of large canid, reportedly double the size of *Canis lupus* and comparable in size to *Aenocyon dirus*, based on several bones from the lead region of contiguous parts of Wisconsin, Iowa, and Illinois (Fig. A1). As early as 1884 and as recently as 2023, confusion has surrounded the taxonomic affiliation and provenance of this taxon. It has been cited as a separate species (Bader & Techter, 1959, 2; Baker, 1920, 353–354; Hay, 1914, 484–487; Hay, 1923, 337), synonymized with *A. dirus* (Caro et al., 2023, 6; Damián, Xiaoming & Ascanio, 2022, 103; Dundas, 1999, Table 1; Kurtén, 1984, 219–220; Kurtén & Anderson, 1980, 171; Merriam, 1912, 220–221; Merriam, 1918, 532; Nowak, 1979, 115; Stevenson, 1978, 179; Tedford, Wang & Taylor, 2009, 114), and synonymized with *C. lupus* (Cope & Wortman, 1884, 11; Hoffmeister, 1989, 37). As far as is known, the species name has not been specifically assigned to other specimens. In regards to provenance, it has been reported as Illinois (Bader & Techter, 1959, 2; Cope & Wortman, 1884, 11), Wisconsin (West & Dallman, 1980), and Blue Mounds, Dane County, Wisconsin (Dundas, 1999, Table 1; Hay, 1923, 342; Kurtén, 1984, 219; Nowak, 1979, 115). Blue Mounds has also been mistakenly reported being located as in Jo Daviess County, Illinois (Anderson, 1905, 12). Despite their commendable efforts, Hay’s (1914, 484–487) and Nowak’s (1979, 115) attempts to resolve the confusion were only partially successful and introduced several errors. Given the direct impact of these issues on the biogeography of *A. dirus*, they were disentangled and reconsidered. *C. mississippiensis* is reassigned to *C. lupus*, with the provenance being the lead region. It is also been reaffirmed that Wyman (1862)’s canids from Blue Mounds are indeed *C. lupus*. The rationale is discussed in the following sections.

### Historical context

In a tome on Wisconsin geology (Hall & Whitney, 1862), Jeffries Wyman (1862) assigned some canid bones from Blue Mounds, Dane County, Wisconsin (WGS 84 43.02N, 89.84W) to *C. lupus* (Fig. A1) In the next chapter, Joseph Leidy (1862, 424) reported on some material from Galena, Jo Daviess County, northwest Illinois (WGS 84 42.42N, 90.43W), 80 km southwest of Blue Mounds and 10 km south of the Wisconsin-Illinois border. He did not mention any canids then nor did he eight years later in another presentation of the fauna, which by that time included two additional taxa, *Megalonyx* and *Bison* (Leidy, 1870, 13). Both locations are within the lead mining region of Wisconsin, Iowa, and Illinois (Knox, 2019a; Walthall, 1981, 27–28), which may explain why Leidy (1862, 424), who was eminently qualified for the task, was asked to inspect the material and report his findings in a volume on Wisconsin geology in the first place. Why Leidy did the original (and subsequent) work, and not Wyman who was also eminently qualified, is unknown. Whatever the reason, Leidy was assuredly interested because Galena and the lead region generally had a track record for yielding extinct Pleistocene fauna, most notably to that point in time, the holotype of *Platygonus compressus* (LeConte, 1848a, 1848b, 1852).

#### Canis mississippiensis

Several years later, Joel Allen identified a new large canid species (*C. mississippiensis*) from bones that “form[ed] part of the collection of mammalian fossils made many years since by Professor J. D. Whitney, from the lead-crevices and superficial strata of the lead region of Wisconsin, Iowa, and Illinois, being a part of those enumerated by the late Professor Jeffries Wyman (1862)” (1876, 47). He did not provide specific provenance for the type-material because he did not know it. However, Allen was certain it was not from Blue Mounds because his bone collection minimally matched what Wyman described 14 years earlier. For example, Wyman’s sample totaled eight specimens, including a broken right humerus, while Allen’s had half that number, including a complete right humerus (Table A1).

Second, Wyman’s bones were indistinguishable from those in *C. lupus*. In contrast, Allen stated his were much larger (than *C. lupus*) after comparing them to those in a *C. lupus* skeleton from Kansas in the Museum of Comparative Zoology, Harvard University (MCZ no. 268) (Fig. A2). He reported the *C. mississippiensis* specimens to be “nearly twice the size” of those in the Kansas animal (1876, 49), and then concluded “it seems impossible that he [Wyman] could have described them as not differing in size from corresponding parts of the gray wolf” (Allen, 1876, 48). That is to say, Allen reasoned Blue Mounds was not the source of his (Allen’s) *C. mississippiensis*. Thus, the taxon was derived from at least one and possibly as many as four locations in the lead region of three contiguous states. The only other canid Wyman (1862, 423) reported from the lead region was a partial *C. latrans* cranium, whose provenance has also been misplaced over the years (Kurtén, 1974, 9; Nowak, 1979, 82).

Allen’s predicament is understandable and his thinking reasonable. His sample differed noticeably from what Wyman described from Blue Mounds and, second, the bones he assigned to *C. mississippiensis* were much larger than the corresponding ones in the Kansas *C. lupus*. However, in using the Kansas *C. lupus* for comparative purposes, he was misled by an exceptionally small individual. The humerus and tibia in *C. mississippiensis* (length 223 and 244 mm, respectively) are unquestionably longer than those in the Kansas animal (length 176 and 200 mm, respectively), and are actually close in size to several relatively small *C. lupus* males and large females (Fig. A2). Measurements are not available for analogous bones from Blue Mounds, however Wyman (1862, 422) stated his specimens were indistinguishable from *C. lupus*.

### Taxonomic and provenance revisions

‘Lead region’ is the most appropriate provenance for Allen’s large canid (*C. mississippiensis*). From whom and when he acquired the specimens is unknown, but obviously it was before publication in 1876. It is also unknown if they are from one or more places. As far as is known, no other canid material relevant to this discussion has been reported from the crevices and fissures around Galena. The provenance of Wyman’s two large canids (based on left mandibles) stays the same (i.e., Blue Mounds, Dane County, Wisconsin).

Allen’s lead region large canid (*C. mississippiensis*) and Wyman’s Blue Mounds large canids are almost certainly *C. lupus*. The lengths of the humerus and tibia in *C. mississippiensis* are about the same as those in smaller *C. lupus* males and larger females (Fig. A2). Both bones are also generally shorter than those in eastern *A. dirus*, especially the humerus. The tibia is about the length as inferred females from Bat Cave and Marlow. Both bones are slightly longer than the majority of those from Rancho La Brea. As well, Wyman identified the Blue Mounds canids as *C. lupus*. Although not stated specifically, it appears he directly compared the specimens to *C. lupus* in his diagnosis. While direct dating will be required to determine the precise temporal position of these specimens, it is notable that all other paleozoological occurrences of *C. lupus* in the region are Holocene in age (Fig. A1), a possibility that Hay (1914, 488) mentioned years ago.

Although the Blue Mounds and lead region canids can no longer be considered as relatively northern occurrences of *A. dirus*, the chances for future regional discoveries of the taxon seem decent. The Driftless Area (Fig. A1) (Knox, 2019b) contains many closed contexts (Alexander, 1980; Day, 1986; Martin, 1965, 93–97) that have not been systematically explored for late Pleistocene vertebrates (but see Foley, 1984; Slaughter & Jones, 2000; Thieling, 1973; Wallace, 2000, 2008; Widga et al., 2012). Additionally, the list of potential prey reported from the region includes *Megalonyx* (Leidy, 1870), *Platygonus* (LeConte, 1852; Palmer, 1974), *Castoroides* (Dallman, 1969), *Bootherium* (McDonald & Ray, 1989, 64); *Cervalces* and *Rangifer* (Josephs, 2005; Long & Yahnke, 2011; West, 1978), and *Mammuthus* and *Mammut* (Boszhardt et al., 1993; Widga et al., 2017). In the end, while fossil evidence is currently absent, it seems probable that *A. dirus* periodically ranged at least as far north as the lead region.

### Kansas *Canis lupus*

The Kansas *C. lupus* skeleton (MCZ no. 268) is an enigma. It was originally cataloged as *C. lupus occidentalis* (= Alaskan wolf, Northwestern wolf) then changed to *C. lupus nubilus* (= buffalo wolf, Great Plains wolf) at an unknown later date. It was collected at Coyote, a now-abandoned railroad colony in Trego County, west-central Kansas, apparently by Allen and two assistants in early January 1872 on their collecting trip to the Great Plains and Rocky Mountains in 1871–1872 (Allen, 1916, 26–27; https://iiif.lib.harvard.edu/manifests/view/ids:49672077).

The humerus and tibia in the Kansas animal are only 5–10 mm longer than those in an exceptionally large adult male *C. latrans* from Iowa County, southwestern Wisconsin (UWZM 27383; live weight 15.5 kg, humerus = 165 mm, tibia = 194 mm), and 15–20 mm shorter than the lightest individual in the Great Lakes *C. lupus* sample (UWZM 27751, adult female; live weight 27.8 kg, humerus = 190 mm, tibia = 217 mm). These comparisons call into question the identification of the Kansas animal. It could actually be a *really* small female *C. lupus*. Alternatively, it may be a misidentified large *C. latrans* (= prairie wolf), or perhaps even a *Canis* hybrid. Whatever the case, it would be interesting to know for certain.

### Provenance of the *Aenocyon dirus* mandible from Logan County, Kansas

Merriam (1912, 221–222, 242–243) reported an *A. dirus* mandible with p2-m3 from the Sheridan formation or beds of Kansas, in the collections of the American Museum of Natural History (FM-10391). Years later, Hibbard (1939, Table 1) questioned this provenance by prefixing it with a question mark (?) (“? Sheridan Formation, Kansas”), possibly because there is no stratum in the state with this name (Tolsted, Swineford & Buchanan, 1986). The American Museum online catalog indicates the specimen is from Logan County, Colorado. Specific provenance is not reported. Online searches for places and geological formations by the same name in Logan County, Colorado, failed to produce any promising leads. However, there is a ghost town named Sheridan in Logan County, Kansas (Snell & Richmond, 1966, 346–347), which incidentally, is located southwest of Sheridan County (Fig. A3). Knowing all is not well and good, the American Museum catalog appears to be incorrect. The most congruent provenance for the mandible is Sheridan (ghost town), Logan County, Kansas. Because the age is unknown, it is placed in the MIS 2-7 bin.

## Supplemental Information

10.7717/peerj.19219/supp-1 Supplemental Information 1 List of supplementary information.

10.7717/peerj.19219/supp-2 Supplemental Information 2 ZooMS methods.

10.7717/peerj.19219/supp-3 Supplemental Information 3 Aeon bone pretreatment procedures.

10.7717/peerj.19219/supp-4 Supplemental Information 4 Metric (mm) and nonmetric data for *Canis lupus* from Wisconsin, Minnesota, and Michigan in the University of Wisconsin Zoological Museum osteology and mammalogy reference collections. Data collected by MGH. 22 adult males and 22 adult females Wisconsin (*n* = 36), Minnesota (*n* = 7), and the Upper Peninsula of Michigan (*n* = 1). The specimens were selected from a collection of 306 males and females of various ages, primarily from Wisconsin.
The measured subsample (*n* = 44) includes the lightest and heaviest individuals of each sex (*n* = 4) in the collection of skeletons from Wisconsin, Minnesota, and the Upper Peninsula of Michigan, plus 40 skeleton selected approximately randomly from this pool.
Age, sex, and taxonomic identity was determined by the collector, often a wolf biologist or game warden. Live weight, date of death, and cause of death data are available for most specimens. Age at death is available for only four individuals. All of the specimens are catalogued as *C. lupus*. Some may be wolf/dog hybrids; ascertaining which ones would require developing genetic information for each specimen. For this work, an adult is considered to be a skeletally mature individual (*i.e*., complete epiphyseal union), which typically aligns with museum records (*i.e*., ‘adult female’).
The same data were recorded for each skeleton. For paired elements and teeth, the left side is the source of the data unless it is missing, articulated, damaged, or pathological, in which case the right side was used. Empty cells indicate the attribute was not recorded because of damage, articulation, or the absence of both sides of the element.
Each skeleton was assigned to a dental age cohort (I-IX) using Stiner’s (1994, Fig. 12.3) eruption and wear sequence for canid m1s.
Measurement protocols derive from several sources: von den Driesch (1976) for the skull, atlas, axis, and scapula; Todd (1987) for the humerus, radius, ulna, femur, and tibia; and Hill (1996) for the calcaneus. Astragalus measurements include: greatest length, lateral length, and width of proximal articular surface. Metapodial measurements include: greatest length, breadth of proximal articular surface, depth of proximal articular surface, midshaft breadth, breadth of distal articular surface, and depth of distal articular surface.

10.7717/peerj.19219/supp-5 Supplemental Information 5 Goldman’s measurements (mm) on *Canis lupus* and *Canis rufus* crania from North America. Data entered by MGH.

10.7717/peerj.19219/supp-6 Supplemental Information 6 Koper’s (*2013, Tables 4 and 6*) measurements (mm) on *Aenocyon dirus* left scapulae and left humeri from Rancho La Brea. Data entered by MGH.

10.7717/peerj.19219/supp-7 Supplemental Information 7 Nigra’s greatest length measurements (mm) on *Aenocyon dirus* left metapodials from Rancho La Brea. Data entered by MGH.

10.7717/peerj.19219/supp-8 Supplemental Information 8 Craniodental measurements (mm) on *Aenocyon dirus* exclusive of Rancho La Brea. Data entered by MGH.

10.7717/peerj.19219/supp-9 Supplemental Information 9 MALDI-TOF spectra for *Aenocyon dirus*, *Canis lupus*, and *Canis latrans*..

10.7717/peerj.19219/supp-10 Supplemental Information 10 *Aenocyon dirus* records in southern North America. Data compiled and entered by MGH.

10.7717/peerj.19219/supp-11 Supplemental Information 11 Measured and calibrated AMS radiometric results on small mammals from Peccary Cave. OxCal 4.4 (Ramsey, 1995; Ramsey, 2001) and IntCal20 (Reimer et al., 2020) used to calibrate the measured radiocarbon age. Data compiled and entered by MGH.

10.7717/peerj.19219/supp-12 Supplemental Information 12 Measured and calibrated AMS radiometric results on *Aenocyon dirus*.. OxCal 4.4 (Ramsey, 1995; Ramsey, 2001) and IntCal20 (Reimer et al., 2020) used to calibrate the measured radiocarbon age. Data compiled and entered by MGH.

10.7717/peerj.19219/supp-13 Supplemental Information 13 Summary statistics.

10.7717/peerj.19219/supp-14 Supplemental Information 14 Wyman’s and Allen’s large canid collections.

10.7717/peerj.19219/supp-15 Supplemental Information 15 Paleozoological occurrences of *Canis lupus* in the Driftless Area and lead mining region. Bogus Cave (BC) (Slaughter, 2001, Table 1). Dwyer Rockshelter (DR) (Speth, Benden & Boszhardt, 2022, 43). Hatfields Cave (HC) (Benn, 1980, Table 17). Lawrence Rockshelter (LR) (Berwick, 1975, Table 3). Mill Pond (MP) (Theler, 1989, Table 5.3). Millville (MV) (Pillaert, 1969, Table 1). Preston Rockshelter (PR) (Theler et al., 2016, Table 2). Pammel Creek (PC) (Theler, 1989, Table 5.3). Quall Rockshelter (QR) (Theler, 2000, Table 2). Raddatz Rockshelter (RR) (Parmalee, 1959, Table 2). Sanders (SA) (Lippold, 1973, Table 1).

10.7717/peerj.19219/supp-16 Supplemental Information 16 Univariate arrays of humerus length and tibia length in *Canis mississippiensis*, *Canis lupus*, and *Aenocyon dirus*. Results for larger samples are displayed as 95% confidence interval box plots with range whiskers. *A. dirus* uses black symbology. *C. mississippiensis* uses red symbology. Paired yellow/blue boxes are females and males, respectively. Labeling is minimized to reduce clutter. Bat and Carroll Cave (Hawksley, Reynolds & McGowan, 1963, Table 2; Hawksley, Reynolds & Foley, 1973, Table 2). Friesenhahn Cave (FC) (Graham, 1976, Table 12; humerus is average of three specimens, tibia is average of four specimens). Marlow (Cifelli, Smith & Grady, 2002, 94). Powder Mill Creek Cave (PMCC) (Galbreath, 1964, 233–234). Rancho La Brea (RLB) (Stock & Lance, 1948). Vero (Sellards, 1919, 154). Table S1. Illustration credit: Matthew G. Hill.

10.7717/peerj.19219/supp-17 Supplemental Information 17 Provenance of the *Aenocyon dirus* mandible from Sheridan, Logan County, Kansas.

## Additional Information and Declarations

### Data Availability

The following information was supplied regarding data availability:

The data is available in the Supplemental Files.
