# Supplementary material for: An update on Aenocyon dirus in the interior of North America: new records, radiocarbon dates, ZooMS spectra, and isotopic data for an iconic late Pleistocene carnivore
Source: PeerJ. 2025 Apr 11;13:e19219. doi: 10.7717/peerj.19219 (PMC11995895; doi:10.7717/peerj.19219)
Supplement: Supplemental Information 1 [file peerj-13-19219-s001.pdf]

Article S1
List of supplementary information

Supplementary Article

Article S1 List of supplementary information. Prepared by MGH.

Article S2 ZooMS methods. Prepared by SAA, MLL, and TAS.

Article S3 Aeon Laboratories, Inc., collagen sample preparation protocols.

Supplementary Table

Table S1 Summary statistics. Prepared by MGH.

Supplementary Data

Data S1 Metric and nonmetric data for *Canis lupus* from Wisconsin, Minnesota, and the Upper Peninsula of Michigan in the University of Wisconsin Zoological Museum (UWZM) osteology and mammalogy reference collections. Data collected by MGH.

The database includes 22 adult males and 22 adult females Wisconsin (n = 36), Minnesota (n = 7), and the Upper Peninsula of Michigan (n = 1). The specimens were selected from a collection of 306 males and females of various ages, primarily from Wisconsin.

The measured subsample (n = 44) includes the lightest and heaviest individuals of each sex (n = 4) in the collection of skeletons from Wisconsin, Minnesota, and the Upper Peninsula of Michigan, plus 40 skeleton selected approximately randomly from this pool.

Age, sex, and taxonomic identity was determined by the collector, often a wolf biologist or game warden. Live weight, date of death, and cause of death data are available for most specimens. Age at death is available for only four individuals. All of the specimens are catalogued as *C. lupus*. Some may be wolf/dog hybrids; ascertaining which ones would require developing genetic information for each specimen. For this work, an adult is considered to be a skeletally mature individual (i.e., complete epiphyseal union), which typically aligns with museum records (i.e., 'adult female').

The same data were recorded for each skeleton. For paired elements and teeth, the left side is the source of the data unless it is missing, articulated, damaged, or pathological, in which case the right side was used. Empty cells indicate the attribute was not recorded because of damage, articulation, or the absence of both sides of the element.

Each skeleton was assigned to a dental age cohort (I-IX) using Stiner's (*1994, Fig. 12.3*) eruption and wear sequence for canid m1s.

Measurement protocols derive from several sources: von den Driesch (*1976*) for the skull, atlas, axis, and scapula; Todd (*1987*) for the humerus, radius, ulna, femur, and tibia; and Hill (*1996*) for the calcaneus. Astragalus measurements

include: greatest length, lateral length, and width of proximal articular surface. Metapodial measurements include: greatest length, breadth of proximal articular surface, depth of proximal articular surface, midshaft breadth, breadth of distal articular surface, and depth of distal articular surface.

Data S2 Goldman's (*1964, Tables 1-6*) measurements on *Canis lupus* and *Canis rufus* (=*Canis niger*) crania from North America. Data entered by MGH.

Data S3 Koper's (*2013, Tables 4 and 6*) measurements on *Aenocyon dirus* left scapulae and left humeri from Rancho La Brea. Data entered by MGH.

Data S4 Nigra's (*1946, Tables 2, 4, 6, 8, 10, 12, 14, and 16*) greatest length measurements (mm) on *Aenocyon dirus* left metapodials from Rancho La Brea. Data entered by MGH.

Data S5 Craniodental measurements on *Aenocyon dirus* exclusive of Rancho La Brea. Data entered by MGH.

Data S6 MALDI-TOF spectra for *Aenocyon dirus*, *Canis lupus*, and *Canis latrans*. Assays by SAA, MLL, and TAS.

Data S7 *Aenocyon dirus* records in southern North America. Data compiled and entered by MGH.

Data S8 Measured and calibrated AMS radiometric results on small mammals from Peccary Cave (*Semken, Graham & Stafford, 2010, Table 4 and supplementary content*). OxCal 4.4 (*Ramsey, 1995; Ramsey, 2001*) and IntCal20 (*Reimer et al., 2020*) used to calibrate the measured radiocarbon age. Data compiled and entered by MGH.

Data S9 Measured and calibrated AMS radiometric results on *Aenocyon dirus*. OxCal 4.4 (*Ramsey, 1995; Ramsey, 2001*) and IntCal20 (*Reimer et al., 2020*) used to calibrate the measured radiocarbon age. Data compiled and entered by MGH.

References

Goldman EA. 1964. *The wolves of North America, part II: classification of wolves*. New York: Dover.

Hill MG. 1996. Size comparison of the Mill Iron site bison calcanea. In: Frison GC, ed. *The Mill Iron site*. Albuquerque: University of New Mexico Press, 231-237.

Koper L. 2013. The anatomical description of the dire wolf (*Canis dirus guildayi*) forelimb from Rancho La Brea. Master's Thesis, Northern Illinois University. https://www.proquest.com/dissertations-theses/anatomical-description-dire-wolf-i-canis-dirus/docview/1502818269/se-2?accountid=10906

Nigra JO. 1946. A statistical study of the metapodials of the dire wolf from the Pleistocene of Rancho La Brea. Master's Thesis, California Institute of Technology. https://resolver.caltech.edu/CaltechTHESIS:02262010-112429311

Ramsey CB. 1995. Radiocarbon calibration and analysis of stratigraphy: the OxCal program. *Radiocarbon* **37(2)**:425-430. https://doi.org/10.1017/S0033822200030903

Ramsey CB. 2001. Development of the radiocarbon calibration program. *Radiocarbon* **43(2A)**:355-363. https://doi.org/10.1017/S0033822200038212

Reimer PJ, Austin WEN, Bard E, Bayliss A, Blackwell PG, Ramsey CB, Butzin M, Cheng H, Edwards RL, Friedrich M, Grootes PM, Guilderson TP, Hajdas I, Heaton TJ, Hogg AG, Hughen KA, Kromer B, Manning SW, Muscheler R, Palmer JG, Pearson C, van der Plicht J, Reimer RW, Richards DA, Scott EM, Southon JR, Turney CSM, Wacker L, Adolphi F, Büntgen U, Capano M, Fahrni SM, Fogtmann-Schulz A, Friedrich R, Köhler P, Kudsk S, Miyake F, Olsen J, Reinig F, Sakamoto M, Sookdeo A, and Talamo S. 2020. The IntCal20 Northern Hemisphere radiocarbon age calibration curve (0–55 cal kBP). *Radiocarbon* **62(4)**:725-757. https://doi.org/10.1017/RDC.2020.41

Semken HA, Graham RW, and Stafford TW. 2010. AMS ^{14}C analysis of late Pleistocene non-analog faunal components from 21 cave deposits in southeastern North America. *Quaternary International* **217(1-2)**:240-255. https://doi.org/10.1016/j.quaint.2009.11.031

Stiner MC. 1994. *Honor among thieves: a zooarchaeological study of neandertal ecology*. Princeton: Princeton University Press.

Todd LC. 1987. Bison bone measurements. In: Frison GC, Todd LC, eds. *The Horner Site: the Type Site of the Cody Cultural Complex*. Orlando: Academic Press, 371-403.

von den Driesch A. 1976. A guide to the measurement of animal bones from archaeological sites. *Bulletin* **1**: Peabody Museum of Archaeology and Ethnology, Harvard University.
