# Supplementary material for: An update on Aenocyon dirus in the interior of North America: new records, radiocarbon dates, ZooMS spectra, and isotopic data for an iconic late Pleistocene carnivore
Source: PeerJ. 2025 Apr 11;13:e19219. doi: 10.7717/peerj.19219 (PMC11995895; doi:10.7717/peerj.19219)
Supplement: Supplemental Information 2 [file peerj-13-19219-s002.pdf]

Article S3
ZooMS methods

For each specimen, acid insoluble collagen was extracted and processed following procedures outlined by Buckley et al. (*2009*) and Brown et al. (*2020*). Between 5 and 10 mg of bone powder obtained from each sample was demineralized in 500 µl of 0.6 M HCl. Following centrifugation at 16000 rpm, acid supernatant was removed and the samples were treated with three washes of 200 µl of 50 mM ammonium bicarbonate, followed by incubation at room temperature in 200 µl of 0.1 M NaOH to remove humic acids. Samples were then treated again with three washes of 200 µl of 50 mM ammonium bicarbonate to return. Following each wash, the supernatant was removed and samples were centrifuged briefly at 16000 rpm. Samples were gelatinized in 100 µl of 50 mM ammonium bicarbonate and placed in an incubator for one hour at 65 °C. Following gelatinization, samples were centrifuged briefly at 16000 rpm and the supernatant was transferred to a new 1.5 mL microcentrifuge tube for trypsin digestion. Collagen extracts were digested in 1 µl of 0.4 µl/µg trypsin overnight for 18 h at 37 °C. After incubation, samples were centrifuged briefly at 16000 rpm and digestion was stopped with 1 µl of 5% (v/v) TFA. Pierce C18 resin 100 µl tips were conditioned by rinsing twice in 100 µl of a conditioning solution of 0.1 % (v/v) TFA in 50 % (v/v) Acetonitrile. The solution was discarded and the tips were rinsed twice in 100 µl of a wash solution of 0.1 % (v/v) TFA in ultrapure water. The sample was then resuspended back and forth over the tip at least 10 times. Following this, the tip was rinsed twice again in 100 µl of the wash solution and discarded. Samples were then eluted in 50 µl of the conditioning solution. Following peptide purification, 1 µl of each sample solution from each C18 resin tip was spotted alongside calibration standards onto a MALDI target plate in triplicate and mixed with 1 µl of α-cyano-4-hydroxycinnamic acid matrix solution. The samples were analyzed using a Sciex 5800 MALDI-TOF mass spectrometer in the Basile Lab at the University of Wyoming. Mass spectra were acquired over a range of 800-3200 m/z.

References

Brown S, Hebestriet S, Wang N, Boivin N, Douka K, Richter KK. **2020**. Zooarchaeology by mass spectrometry (ZooMS) for bone material - acid soluble protocol. *protocolsio*. https://dx.doi.org/10.17504/protocols.io.bf5bjq2n

Buckley M, Collins MJ, Thomas-Oates J, Wilson JC. **2009**. Species identification by analysis of bone collagen using matrix-assisted laser desorption/ionisation time-of-flight mass spectrometry. *Rapid Communications in Mass Spectrometry* **23**(**23**):3843-3854. https://doi.org/10.1002/rcm.4316
