# Supplementary material for: An update on Aenocyon dirus in the interior of North America: new records, radiocarbon dates, ZooMS spectra, and isotopic data for an iconic late Pleistocene carnivore
Source: PeerJ. 2025 Apr 11;13:e19219. doi: 10.7717/peerj.19219 (PMC11995895; doi:10.7717/peerj.19219)
Supplement: Supplemental Information 3 [file peerj-13-19219-s003.pdf]

Sample Preparation

weigh and record initial mass

mechanically clean as needed to remove surface contamination and chalky bone material.

Use stainless-steel brush, or grind with rotary carbide tooling to remove all visible surface contamination and select for densest fraction of sample. Note: it may be impossible to remove extraneous material from bone pores. For tooth samples, dentin is the preferred fraction. Although enamel is removed during demineralization, and small bits are not detrimental to the analysis, it is better to remove as much of it as possible at this point, as it increases demineralization time and the amount of acid needed.

select ~5 mg representative sample, powder to < 0.3 mm, for CHN elemental analysis

EA sample may be powdered by grinding, e.g., with Dremel & carbide tile/grout cutter

record mass, wait for results

if either **%N < 0.15%** or **C:N > 11**, recommend terminating analysis

select a 75..2400 mg lab sample for demineralization, record mass

use *bone & tooth.ods* to estimate quantity based on elemental analysis

crush ***(do not grind)*** to < 0.3 mm powder

first crush to < 0.6 mm using SS mortar and pestle, then finish crushing to < 0.3 mm

record mass of powdered lab sample

demineralize in ≥ 12 mL 0.5 N HCl per 100 mg sample at room temperature under moderate vacuum

Loosely cap or cover the sample centrifuge tube or beaker. Do not seal vessel. Degas in unheated vacuum chamber, at sufficient vacuum to nearly boil. Control the depressurization rate to avoid foaming. Maintain a vacuum pressure that forms frequent small bubbles and pops large surface bubbles, but does not produce a “rolling” boil. After the first 5 minutes, when most of the mineral is dissolved, briefly return pressure to atmosphere and gently suspend any solids stuck to the tube/beaker wall above the liquid surface. Do not agitate or entrain air into the solution. Simply tilt and rotate the sample container, using the surface tension to draw the particles into the liquid. Then continue degassing until the sample is demineralized (typically about 15 minutes more). Completion is detected when the vacuum required to produce the small bubbles causes the solution to boil.

rinse 5 times with pH3 water (0.001 N HCl)

{ Note: A*t pH ≥ 4, collagen residues typically clump and stick to the centrifuge tube wall. Tiny bits of ferrous material can be removed by sliding a neodymium magnet along the outside of the centrifuge tube to draw the magnetic particles up the wall, near to the top of the tube, above the surface of the liquid; the material sticks there. Then, remove the cap and attract the material to the magnet lowered into the tube but not touching anything. }*

immerse 1 h in ~20 mL 0.1 N NaOH at room temperature
cap tightly, shake vigorously if needed to disperse clumps
and suspend particles adhered to centrifuge tube wall, then
gently agitate until bubbles disappear and sample particles submerge
centrifuge; discard supernatant

rinse 5 times with pH3 water (0.001 N HCl)

dry in vacuum chamber at room temperature (~4..10 h, depending on the number of samples)
Use LN-cooled water trap for the first 2 h. After LN is removed and water trap is dry, determine dryness by turbopump backing pressure (minimal change when chamber is isolated).
Very wet samples may be frozen first to prevent boiling and splashing the sample.
record mass (residual "collagen")

This concludes the recommended portion of the wet-chemistry pretreatment. If the collagen absolutely must be denatured to gelatin, then continue as follows;

select ~20 mg residual collagen for gelatinization (more if sample is badly contaminated); record mass

denature to gelatin 4..40 h in 1.7 mL pH 2 water (0.01 N HCl) at 58..62 °C

use tall hot block and cap centrifuge tube tightly to prevent evaporation loss

Solvent pH is critical. *Below pH 2, samples dry poorly, to a crystalline structure with suboptimal elemental analysis (neutralization leaves undesirable salt). At pH ≥ 3, samples do not denature completely.*

*{ **Note**: this step is known to increase the radiocarbon activity of some samples, particularly the Snowmass bone blank Aeon 55.1. Avoid gelatinization and subsequent wet chemistry on this and similar samples. }*

centrifuge to concentrate residual solids, re-cap tubes and return to hot block

prepare a filtered syringe for each sample

<u>if ultrafiltration is not required</u>

filter the gelatin into a new 1.5 mL centrifuge tube

heat an empty pre-cleaned 25 mL glass beaker to 60 °C

remove the prepared syringe filter and draw ~2 mL air into syringe

decant the warm gelatin into the heated beaker

Do not place heated beaker directly onto a cool work surface.

draw the gelatin from the beaker into the syringe

immediately re-attach the syringe filter and dispense the syringe contents through the filter into a new 1.5 mL centrifuge tube

<u>if ultrafiltration is required</u>

prepare an ultrafilter for each sample requiring ultrafiltration

mark the ultrafilter and its cap with the sample ID

filter the gelatin into the ultrafilter

heat an empty pre-cleaned 25 mL glass beaker to 60 °C

remove the prepared syringe filter and draw ~2 mL air into syringe

decant the warm gelatin into the heated beaker

Do not place heated beaker directly onto cool tabletop.

draw the gelatin from the beaker into the syringe

immediately re-attach the syringe filter and dispense the syringe contents through the filter into the ultrafilter

add 0.1 µm filtered pH3 water to raise volume to 6 mL

ultrafilter:

centrifuge 15..90 minutes to 1 mL < volume < 1.5 mL *(cool centrifuge with fan)*

discard filtrate from bottom of ultrafilter *(the high MW gelatin is the filtrand/retentate)*

transfer the purified gelatin from the ultrafilter into a new 1.5 mL centrifuge tube

dry in vacuum centrifuge/concentrator @ ≤ 60 °C (~4..6 h)

start centrifuge before applying vacuum (or sample may bubble out of tube)

an *LN-cooled water trap is needed for at least the first 1 or 2 h*

do not stop concentrator centrifuge rotor before drying is complete

isolate evacuated concentrator chamber from system to service water trap

determine dryness by turbopump backing pressure

after LN is removed and water trap is emptied and dry

weigh sample and record mass

select 2..3 mg (2.8 mg nominal) for 14C analysis

expected C yield: ~37-45% (625 °C fraction after discarding 150 °C fraction)

select ~3 mg for EA to assess gelatin quality

(optional) select 1.2..1.4 mg for solid sample $\delta^{15}N$ & $\delta^{13}C$ analysis by IRMS

<u>Implements and materials preparation</u>

Prepare filtered syringes and ultrafilters only immediately before use.

<u>filtered syringe preparation</u>
fill and discharge 5 mL UPW through new 5 or 10 mL syringe
draw 1..2 mL air and > 2 mL UPW into syringe
connect a 0.1 μm nylon syringe filter to the syringe
expel syringe contents vertically downward through filter to waste

<u>ultrafilter preparation (Vivaspin 6, 30 kD MWCO)</u>
(Wear gloves when handling ultrafilters until after sonication.)
centrifuge 6 mL UPW through ultrafilter and discard filtrate (~6 minutes), twice
dust exterior of ultrafilter with compressed air
sonicate ultrafilter (but not the tube or cap) at least 1 h in > 100 mL UPW per ultrafilter
centrifuge 6 mL UPW through ultrafilter and discard filtrate
Do not allow the ultrafilter to dry out once it has been prepared.
