# Supplementary material for: An update on Aenocyon dirus in the interior of North America: new records, radiocarbon dates, ZooMS spectra, and isotopic data for an iconic late Pleistocene carnivore
Source: PeerJ. 2025 Apr 11;13:e19219. doi: 10.7717/peerj.19219 (PMC11995895; doi:10.7717/peerj.19219)
Supplement: Supplemental Information 13 [file peerj-13-19219-s013.pdf]

Table S1
Summary statistics

Taxon	Group	Element	Attribute	Sex	n	min (mm)	max (mm)	95%CI (mm)	95%CI (mm)	mean (mm)	Source
Aenoycon dirus	Rancho La Brea	cranium	post-orbital constriction	male	62	43.5	54.4	49.8	48.8	49.3	Nowak, 1979, Appendix B
Canis lupus	Great Lakes	cranium	post-orbital constriction	female	20	36.6	46.3	41.4	39.3	40.3	this study, Data S1
Canis lupus	Great Lakes	cranium	post-orbital constriction	male	18	38.3	45.3	43.0	40.9	41.9	this study, Data S1
Canis lupus	North America	cranium	post-orbital constriction	female	123	30.4	46.9	40.1	39.0	39.6	Goldman, 1964; Data S2
Canis lupus	North America	cranium	post-orbital constriction	male	176	31.4	49.5	41.9	40.8	41.3	Goldman, 1964; Data S2
Canis rufus	North America	cranium	post-orbital constriction	female	46	31.0	41.5	37.3	35.9	36.6	Goldman, 1964; Data S2
Canis rufus	North America	cranium	post-orbital constriction	male	64	31.4	42.1	37.4	37.1	36.7	Goldman, 1964; Data S2
Aenoycon dirus	Rancho La Brea	cranium	post-orbital processes	unknown	62	73.4	100.0	84.3	82.6	83.5	Nowak, 1979, Appendix C
Canis lupus	Great Lakes	cranium	post-orbital processes	female	20	55.0	75.7	63.0	58.5	60.7	this study, Data S1
Canis lupus	Great Lakes	cranium	post-orbital processes	male	18	58.6	72.5	65.5	61.8	63.7	this study, Data S1
Aenoycon dirus	Rancho La Brea	P4	mesiodistal length	unknown	62	28.7	35.3	32.3	31.2	31.8	Nowak, 1979, Appendix C
Canis lupus	Great Lakes	P4	mesiodistal length	female	21	20.8	24.7	23.3	22.4	22.9	this study, Data S1
Canis lupus	Great Lakes	P4	mesiodistal length	male	20	22.7	25.6	24.7	23.9	24.3	this study, Data S1
Canis lupus	North America	P4	mesiodistal length	female	122	21.0	26.5	24.3	23.9	24.1	Goldman, 1964; Data S2
Canis lupus	North America	P4	mesiodistal length	male	174	22.5	30.6	26.1	25.7	25.9	Goldman, 1964; Data S2
Canis rufus	North America	P4	mesiodistal length	female	46	19.1	28.8	22.7	21.7	22.2	Goldman, 1964; Data S2
Canis rufus	North America	P4	mesiodistal length	male	64	20.1	25.2	23.1	22.5	22.8	Goldman, 1964; Data S2
Aenoycon dirus	Maricopia	m1	mesiodistal length	unknown	17	33.5	37.0	36.0	34.0	35.0	Nowak, 1979, Appendix C
Aenoycon dirus	McKittrick	m1	mesiodistal length	unknown	15	33.7	37.0	36.0	34.6	35.3	Nowak, 1979, Appendix C
Aenoycon dirus	Rancho La Brea	m1	mesiodistal length	unknown	73	31.8	38.5	34.7	33.8	34.3	Nowak, 1979, Appendix C
Canis lupus	Great Lakes	m1	mesiodistal length	female	21	24.1	28.9	27.1	25.9	26.5	this study, Data S1
Canis lupus	Great Lakes	m1	mesiodistal length	male	21	26.0	30.1	28.4	27.4	27.9	this study, Data S1
Canis lupus	North America	m1	mesiodistal length	female	118	24.9	30.9	27.9	27.4	27.6	Goldman, 1964; Data S2
Canis lupus	North America	m1	mesiodistal length	male	173	24.9	33.8	29.7	29.2	29.4	Goldman, 1964; Data S2
Canis rufus	North America	m1	mesiodistal length	female	46	20.0	27.8	24.9	24.0	24.5	Goldman, 1964; Data S2
Canis rufus	North America	m1	mesiodistal length	male	64	22.3	27.9	25.6	24.9	25.2	Goldman, 1964; Data S2

Aenoycon dirus	Rancho La Brea	scapula	length of glenoid fossa	unknown	26	34.0	42.2	38.2	36.4	37.3	Koper, 2013, Table 4; Data S3
Canis lupus	Great Lakes	scapula	length of glenoid fossa	female	22	21.4	35.5	28.7	24.8	26.7	this study, Data S1
Canis lupus	Great Lakes	scapula	length of glenoid fossa	male	22	23.0	38.6	32.5	27.2	29.8	this study, Data S1
Aenoycon dirus	Rancho La Brea	humerus	greatest length	unknown	496	194.0	241.0	218.1	217.7	217.9	Stock and Lance, 1948
Canis lupus	Great Lakes	humerus	greatest length	female	20	190.0	235.0	216.6	207.4	212.0	this study, Data S1
Canis lupus	Great Lakes	humerus	greatest length	male	22	213.0	241.0	231.8	225.6	228.7	this study, Data S1
Aenoycon dirus	Rancho La Brea	humerus	midshaft breadth	unknown	17	16.9	22.0	19.8	18.2	19.0	Koper, 2013, Table 6; Data S3
Canis lupus	Great Lakes	humerus	midshaft breadth	female	21	13.5	16.8	15.5	14.6	15.1	this study, Data S1
Canis lupus	Great Lakes	humerus	midshaft breadth	male	22	14.9	17.7	16.8	16.1	16.5	this study, Data S1
Aenoycon dirus	Rancho La Brea	radius	greatest length	unknown	740	183.0	233.0	209.5	209.3	209.4	Stock and Lance, 1948
Canis lupus	Great Lakes	radius	greatest length	female	22	195.0	233.0	218.5	210.4	214.5	this study, Data S1
Canis lupus	Great Lakes	radius	greatest length	male	22	213.0	246.0	232.6	225.1	228.8	this study, Data S1
Aenoycon dirus	Rancho La Brea	metacarpal V	greatest length	unknown	1,298	62.0	87.0	74.0	73.6	73.8	Nigra, 1946, Table 8; Data S4
Canis lupus	Great Lakes	metacarpal V	greatest length	female	21	68.0	90.0	80.9	76.4	78.6	this study, Data S1
Canis lupus	Great Lakes	metacarpal V	greatest length	male	22	78.0	92.0	85.6	82.8	84.2	this study, Data S1
Aenoycon dirus	Rancho La Brea	tibia	greatest length	unknown	687	208.0	257.0	233.6	233.4	233.5	Stock and Lance, 1948
Canis lupus	Great Lakes	tibia	greatest length	female	21	217.0	263.0	241.7	231.2	236.8	this study, Data S1
Canis lupus	Great Lakes	tibia	greatest length	male	22	231.0	266.0	255.8	248.2	252.0	this study, Data S1
Aenoycon dirus	Rancho La Brea	calcaneus	greatest length	unknown	11	60.1	72.4	67.6	62.6	65.1	this study, Table 5
Canis lupus	Great Lakes	calcaneus	greatest length	female	22	54.0	63.0	58.5	56.5	57.5	this study, Data S1
Canis lupus	Great Lakes	calcaneus	greatest length	male	22	55.0	66.0	63.0	61.0	62.0	this study, Data S1
