# Supplementary material for: An update on Aenocyon dirus in the interior of North America: new records, radiocarbon dates, ZooMS spectra, and isotopic data for an iconic late Pleistocene carnivore
Source: PeerJ. 2025 Apr 11;13:e19219. doi: 10.7717/peerj.19219 (PMC11995895; doi:10.7717/peerj.19219)
Supplement: Supplemental Information 14 [file peerj-13-19219-s014.docx]

| Blue Mounds  Jefferies Wyman | lead region  Joel Allen |
| --- | --- |
| 1. left mandible with m2-3 2. complete left mandible, edentulous 3. right maxilla fragment with M1 4. broken right humerus 5. broken right femur 6. broken right tibia 7. complete left tibia 8. complete metatarsal | 1. complete right humerus 2. distal right femur 3. complete tibia 4. nearly complete right tibia |
