# Supplementary figures and images for: An update on Aenocyon dirus in the interior of North America: new records, radiocarbon dates, ZooMS spectra, and isotopic data for an iconic late Pleistocene carnivore
Source: PeerJ. 2025 Apr 11;13:e19219. doi: 10.7717/peerj.19219 (PMC11995895; doi:10.7717/peerj.19219)

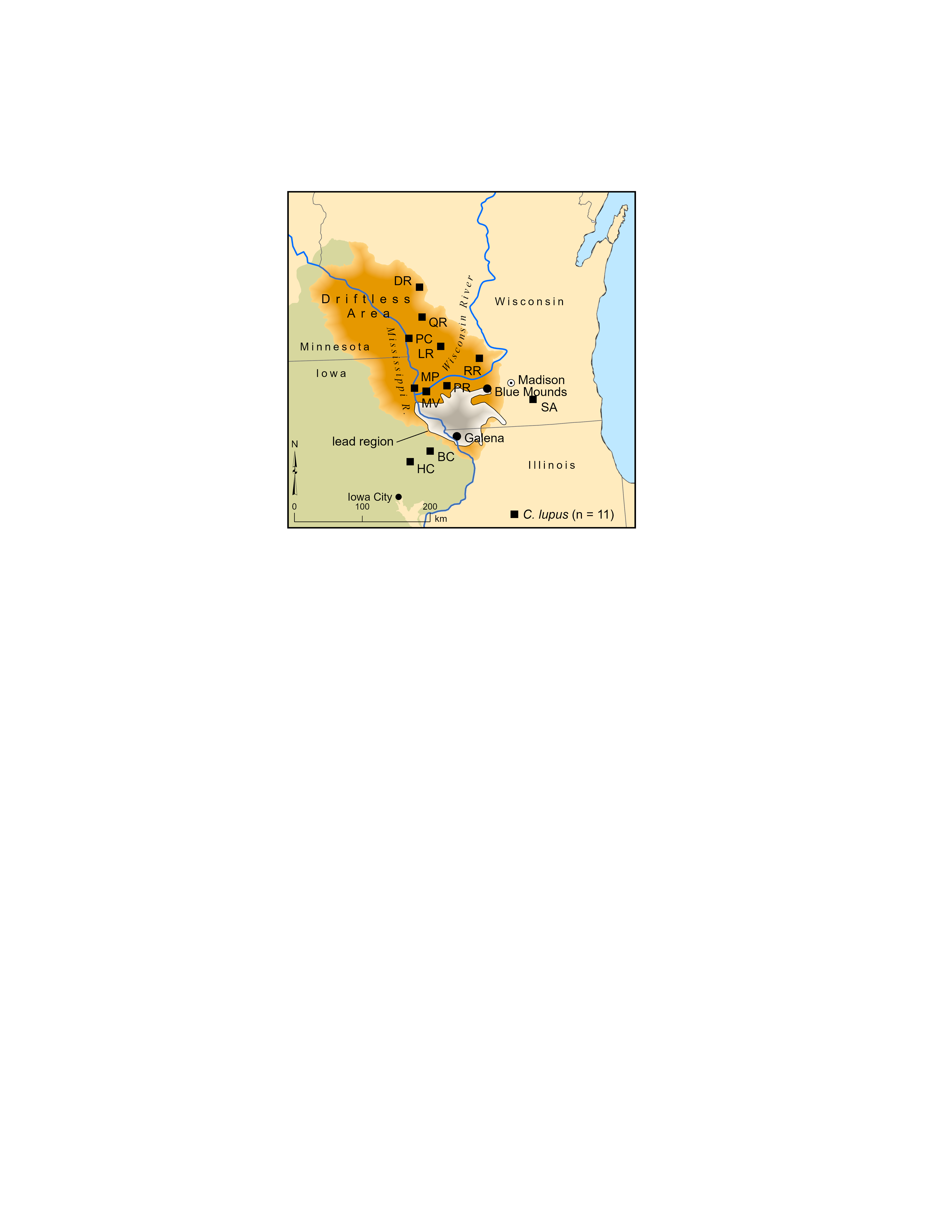

Supplement: Supplemental Information 15 — Bogus Cave (BC) (Slaughter, 2001, Table 1). Dwyer Rockshelter (DR) (Speth, Benden & Boszhardt, 2022, 43). Hatfields Cave (HC) (Benn, 1980, Table 17). Lawrence Rockshelter (LR) (Berwick, 1975, Table 3). Mill Pond (MP) (Theler, 1989, Table 5.3). Millville (MV) (Pillaert, 1969, Table 1). Preston Rockshelter (PR) (Theler et al., 2016, Table 2). Pammel Creek (PC) (Theler, 1989, Table 5.3). Quall Rockshelter (QR) (Theler, 2000, Table 2). Raddatz Rockshelter (RR) (Parmalee, 1959, Table 2). Sanders (SA) (Lippold, 1973, Table 1). [file peerj-13-19219-s015.png]

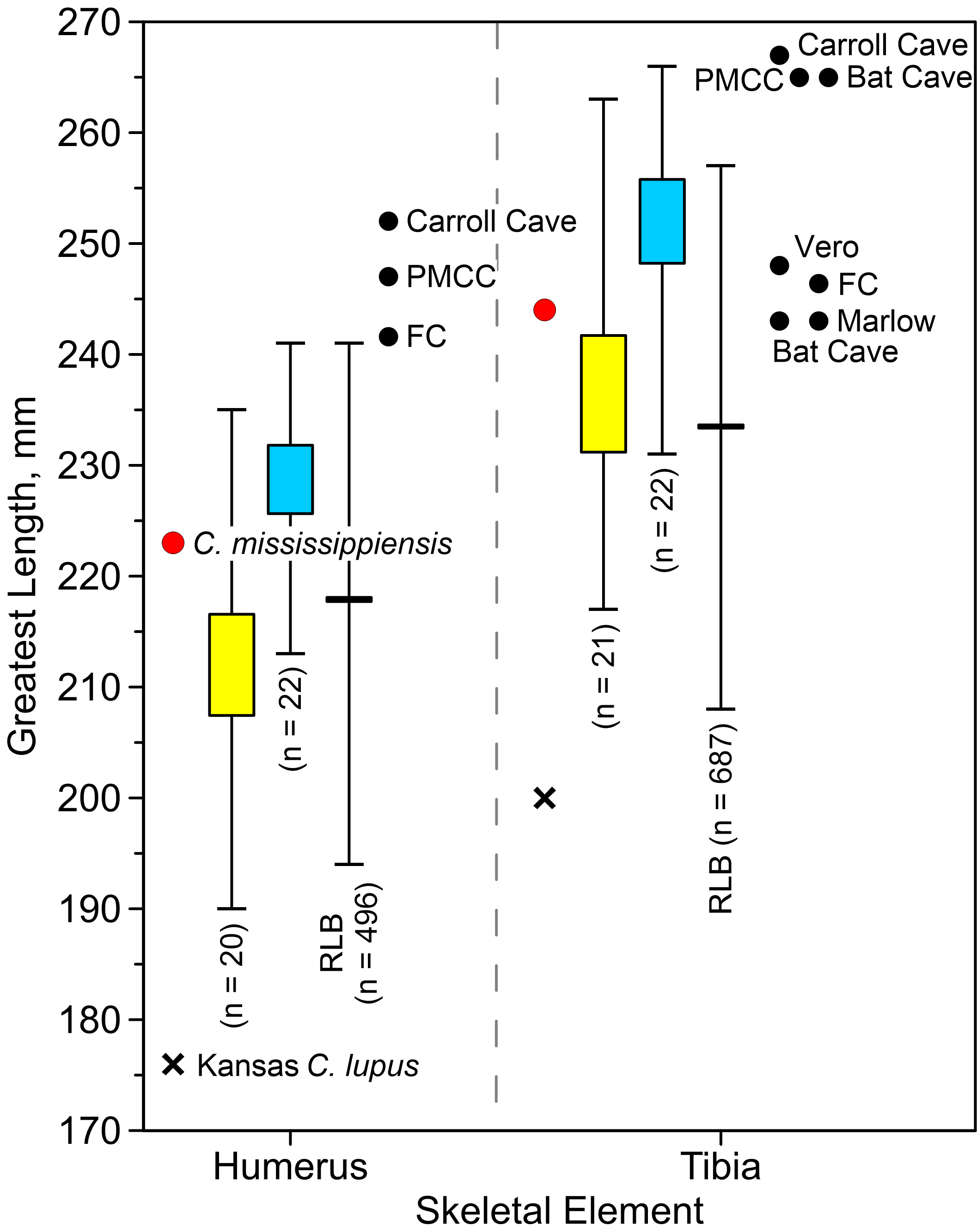

Supplement: Supplemental Information 16 — Results for larger samples are displayed as 95% confidence interval box plots with range whiskers. A. dirus uses black symbology. C. mississippiensis uses red symbology. Paired yellow/blue boxes are females and males, respectively. Labeling is minimized to reduce clutter. Bat and Carroll Cave (Hawksley, Reynolds & McGowan, 1963, Table 2; Hawksley, Reynolds & Foley, 1973, Table 2). Friesenhahn Cave (FC) (Graham, 1976, Table 12; humerus is average of three specimens, tibia is average of four specimens). Marlow (Cifelli, Smith & Grady, 2002, 94). Powder Mill Creek Cave (PMCC) (Galbreath, 1964, 233–234). Rancho La Brea (RLB) (Stock & Lance, 1948). Vero (Sellards, 1919, 154). Table S1. Illustration credit: Matthew G. Hill. [file peerj-13-19219-s016.png]

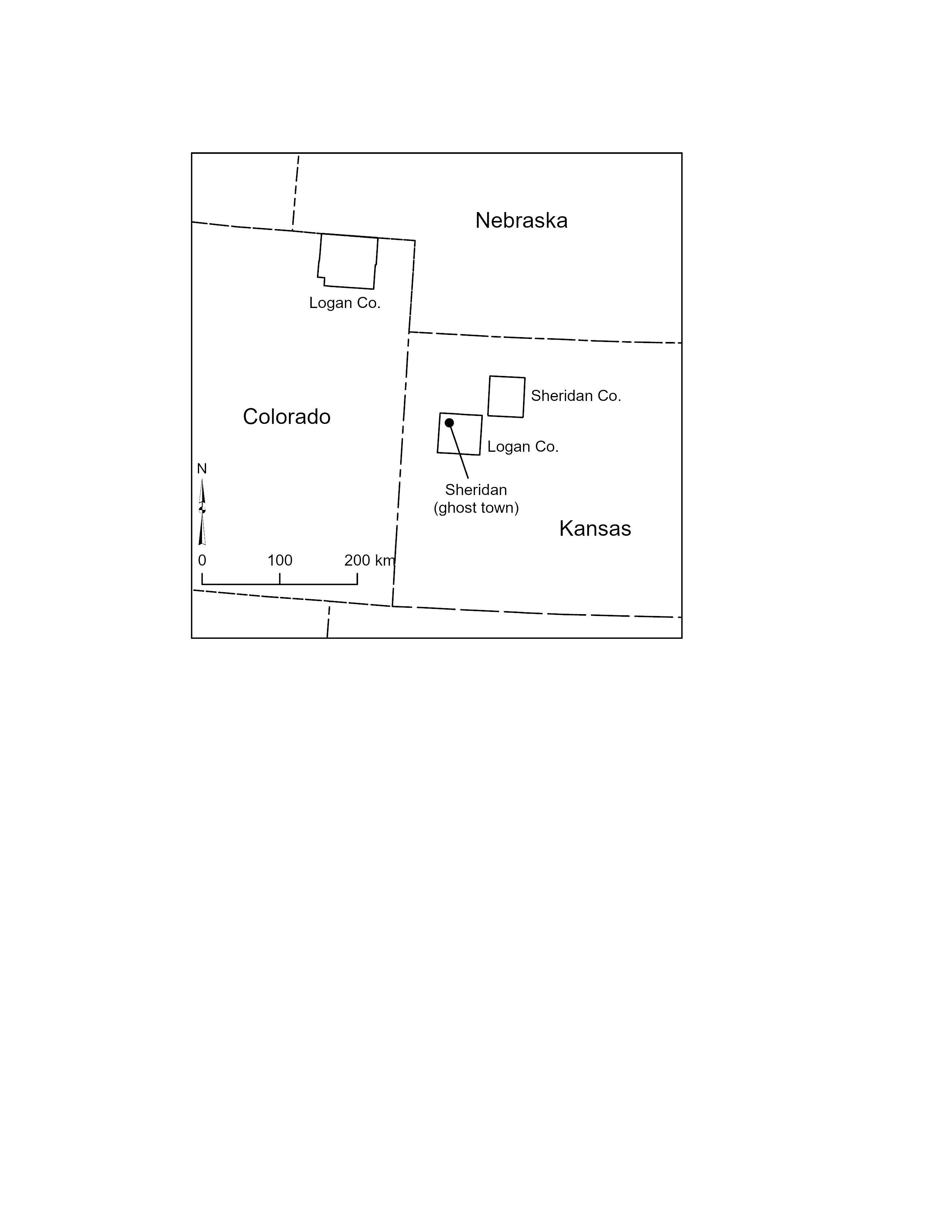

Supplement: Supplemental Information 17 [file peerj-13-19219-s017.png]
